# Moonrat II: A portable incubator

**DOI:** 10.1016/j.ohx.2026.e00849

**Published:** 2026-10-31

**Authors:** Robert L. Read, Melanie Y. Laporte, Forrest “Lee” Erickson, Horacio García–Vázquez, Silvia A. Casillas, L. Enrique Ruiz–Fernández

**Affiliations:** a Public Invention, 1709 Norris Dr., Austin, TX 78704, United States of America; b Columbia University, 116th and Broadway, New York, NY 10027, United States of America; c Camino del Bosque 733, Bosques del Poniente, Tercer Sector, Santa Catarina, Nuevo Leon 66362, Mexico; d Department of Automatic Control, Centro de Investigación y de Estudios Avanzados del I.P.N. (Cinvestav), Av. del Bosque 1145, Zapopan, Jalisco, CP 45019, Mexico; e Centro Universitario de los Valles, Universidad de Guadalajara, Department of Computer Science and Engineering, Carretera Guadalajara–Ameca Km. 45.5, Ameca, Jalisco, CP 46600, Mexico

**Keywords:** Petrifilm™, Incubator, Portable, Microbial analysis, Field testing, Open-source

## Abstract

Incubation of biological samples in the field, far away from a laboratory, requires a portable, rugged, battery-powered incubator. The Moonrat II is a $225 open-source software and open-source hardware incubator constructed from a stainless steel vacuum flask and a microcontroller that can be powered by widely used external lithium-ion battery packs. It can be manufactured with modest skills and readily available tools. A three-button interface and an OLED display provide configurability and an ongoing graph of temperature to provide confidence in the incubation. A complete one-minute-resolution history of the internal temperature during an experiment can be downloaded via the serial port after the experiment. Measurements of the spatial consistency of the temperature are provided, as well as temperature stability over 48 h. A formula for battery life is provided based on power consumption measurements at room temperature and at refrigeration temperature. A statistical comparison with a commercial desktop incubator using 3M E. coli Petrifilms™ was performed, demonstrating a mean ratio of 1.076 between the two incubators. Due to the high variability of the measurements, this was statistically insufficient to establish equivalence or a difference between the incubators.

## Graphical abstract

**Figure:**
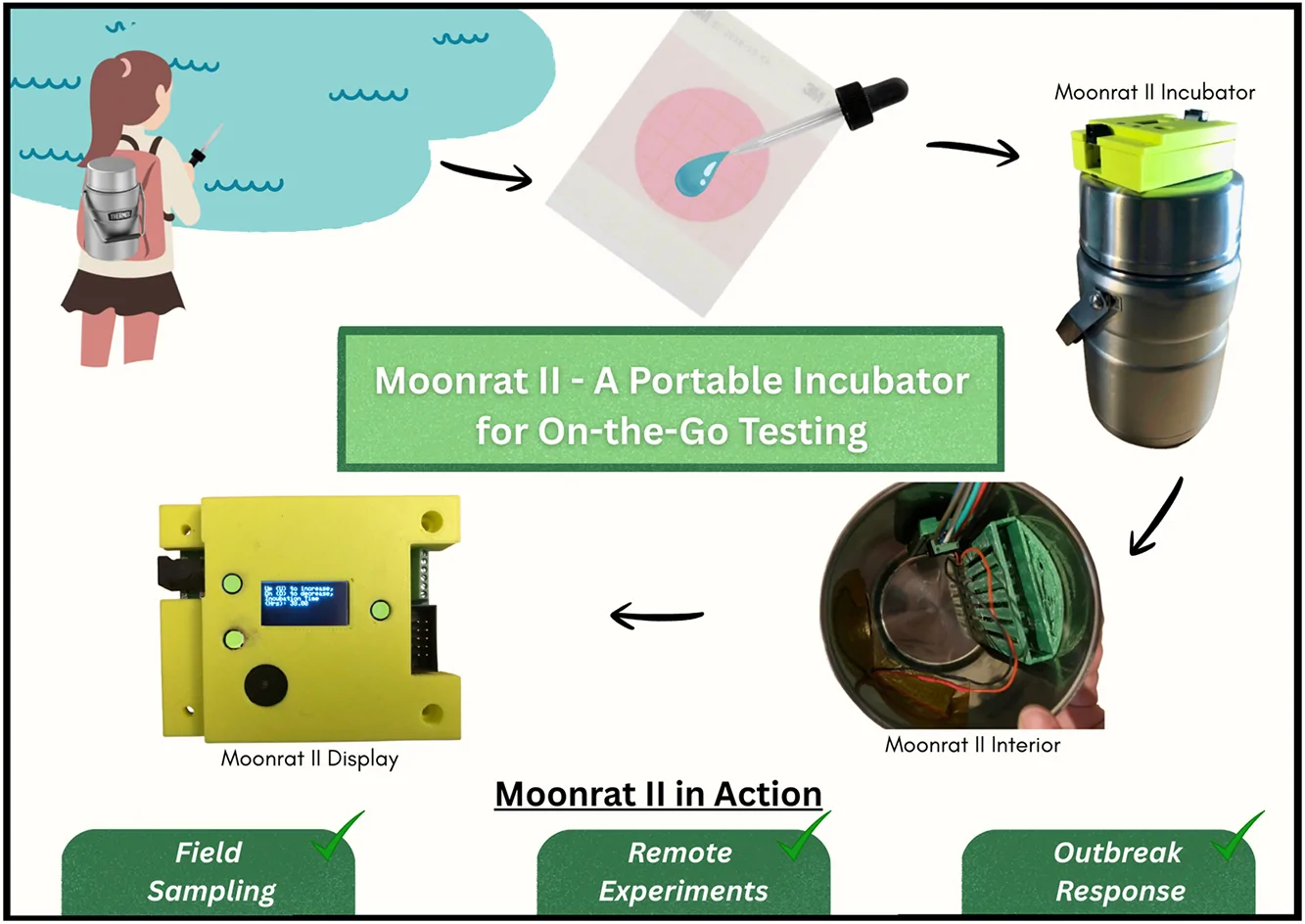

## Specifications table

**Table:** 

Hardware name	Moonrat II Portable Incubator
Subject area	Public Health, Sanitation, Environmental, Planetary and Agricultural Sciences, Public Health
Hardware type	Biological sample handling and preparation

Closest commercial analog	This hardware prototype could potentially replace conventional, larger, and less portable incubators typically used in laboratory settings for microbial analysis using 3M Petrifilm™.

Open source license	*CERN Open Hardware Licence Version 2 - Strongly Reciprocal*
Cost of hardware	$225.00 USD
Source file repository	https://doi.org/10.5281/zenodo.20531233
OSHWA certification UID	US002666

## 1 Hardware in context

Incubation of biological samples at controlled, slightly elevated temperatures for specific periods of time is essential for many kinds of biological tests. Not least of these is the detection of E. coli, a marker for fecal contamination and water-borne diseases that kill around 395,000 children every year [1]. There are many other materials, including the eggs of vertebrates, that require incubation. Keeping a box warm is simple and easy if you have a laboratory with reliable power. However, laboratory incubators cost approximately $500–$1500 US dollars, require reliable 110VAC power, and weigh about 15–40 pounds. It is often useful and sometimes required to begin incubation soon after sample collection in the field. For example, the US EPA [2] states that water quality technicians should immediately cool drinking water samples to temperatures below 10°C and have a holding time (the maximum time before laboratory analysis and incubation at elevated temperature should begin) of 30 h. In rural locations far from laboratories and separated by rugged terrain, these guidelines can be so impractical that they preclude water sample analysis altogether. Even if possible, transporting samples to a laboratory could delay crucial decision making. The Moonrat II solves this problem. A rugged, portable, battery-powered incubator, the Moonrat II ensures that the sample has been held at the correct temperature for the correct period of time. (*Moonrat* is slang in the state of Texas for *Didelphis virginiana*, a gray, nocturnal, rat-shaped, naked-tailed opossum, the only North American marsupial which “incubates” its young in a warm pouch.)

One of the authors volunteers for Engineers Without Borders USA [3], which works with local communities worldwide to provide safe drinking water [4]. As part of this work, EWB USA often measures the bacterial contamination of groundwater and well water both before and after treatment. Preventing water-borne disease is a primary concern. Measuring E. coli in water does not make it clean, but is a valuable practical guide to water remediation and relative safety.

It is a happy accident that E. coli is more detectable in water than pathogenic organisms, and a reliable, if imperfect, marker for dangerous fecal contamination. However, to reach detectable levels, thermophilic E. coli must be cultured at 35°C (typically) and receive nutrients for 24 to 48 h. To do this reliably requires a temperature-controlled incubator. Unfortunately, EWB USA typically works in rural communities far from both laboratories and reliable electricity with which to incubate this culture.

There are countless other uses for biological incubation that the authors are not expert enough to report on. We imagine that even herpetologists and ornithologists may someday use the Moonrat II.

### 1.1 Related research

Having worked in this area for some time, it is clear that many people have recognized the problem of rural incubation, particularly with respect to bacterial water quality analysis [5]. This journal has already reported on perhaps the most similar example of a portable incubator, a device which includes multiple incubation chambers. It differs from the Moonrat II, however, by using a simple digital thermostat instead of our microcontroller system. It can also connect a smart phone to monitor the samples as they are incubated, which is beyond the scope of the Moonrat II. However, no single dominant solution for rural incubation has emerged, perhaps because the demand is relatively low and it seems unlikely to motivate a for-profit business. The notable systems of previous incubator functionality, such as [5], include:

• [6] a comprehensive test of a cooler-based design, although it does not provide an open-source version of the design.
• [7] a portable incubator that uses a camera to detect E. coli and coliform bacteria, powered by a car battery.
• [8] versatile use of an egg incubator (presumably non-portable).
• [9] a portable, solar-powered incubator that does not seem to be open.
• [10] a portable open-source system for under €1250.
• [11] a fuzzy controller similar to the one that we optionally implemented.
• [12] an inexpensive laboratory incubator.

Unlike traditional laboratory-based benchtop equipment [13], our incubator is designed to be portable and used in remote and rural environments. Thus, the Moonrat II will provide reliable microbial cultivation results outside of a laboratory setting.

Our innovation aims to address the limitations of current desktop incubators by offering a compact, portable solution that operates independently of grid power, enabling a variety of biological experiments and assays to be performed directly “in the field”.

The Moonrat II is a significant improvement on our previous design (the Armadillo), which was based on a much larger, insulated cooler [14]. While it was effectively utilized in Iraq, the user found it large and cumbersome. To address this shortcoming, the Moonrat II now employs a vacuum flask, which is smaller and offers equal or superior insulation. The Moonrat II improves the vacuum flask and software usability of the Moonrat I.

## 2 Hardware description

Like [5], we chose to use a vacuum flask as an incubation chamber because it is more heat efficient and smaller than an insulated chamber. The cited incubator is close to our work, but differs in its use of a simpler thermostat, whereas we chose a microcontroller. The microcontroller allows us to present a temperature graph and log. This will give the user confidence that the Moonrat II maintained its target temperature for the entire period. We chose an Arduino Minima as a widely available microcontroller, which is more than adequate for the Moonrat II’s minimal computational needs. Having a sufficient static memory, it allows us to log temperatures every minute. The graph drawn backwards in time thus assures the operator of the sample’s temperature history.

External batteries are a convenient energy source for the Moonrat II, as it can use common lithium ion rechargeable battery packs. A typical incubation course requires, at most, two such batteries. One battery may charge, using either a solar charger or other source, while the other battery is working. Alternatively, one may simply take several batteries into the field.

The total size of our system (28 cm high by 13 cm by 12.5 cm) easily fits within a small backpack or tote and weighs about five pounds.

Also following [5], we chose to place our control electronics in an enclosure which is attached to, rather than integrated into, the incubation chamber. This allows a researcher, in theory, to use the same controller with different incubation chambers for different kinds of media, such as 3M Petrifilms™  , 100 mm petri dishes, 125 mm petridishes, or even test-tube racks. A large incubation chamber would probably have a greater typical thermal loss than the 1.1 W that we observe with our Thermos Big Boss™  food jar at room temperature, but this just necessitates larger batteries.

A small “heater board” attaches to the main controller via a 6-wire ribbon cable. The heater board is a small printed circuit board assembly which fits directly into the incubation chamber. Two simple mylar joule-heating cloths in series and the digital thermometer both attach to the heater board, which is glued to the inside of the lid. The wires to the thermometer and the heater cloth are long enough to allow the lid to unscrew with two complete rotations.

In order to produce a uniform temperature within the incubator, we were forced to create an internal aluminum cylinder. This cylinder can be made with a hack saw, tin snips from an aluminum tube, and sheets of aluminum. We wrapped heating cloths around this cylinder and taped them to the cylinder body, ensuring that a portion of the cloths remained in contact with the end discs. Since the aluminum conducts heat, this design provides measured uniform heat throughout the entire cylinder. Additionally, since the cylinder and the heating cloths are inside the vacuum flask, the system remains heat-efficient. Unfortunately, the volume of the incubation chamber is about 30%.

If a user desires a different incubation chamber, they must use an alternative means of attaching the heater board, the heater, and the thermometer inside the incubation chamber. Simple hot-melt glue would probably work in most cases. The heating cloth must not directly touch whatever sample holders the user chooses, and the digital thermometer should not touch the heating cloth.

The controller board has two electrical ports. The first is a standard 12 V 2.1 mm barrel jack that takes a plug from a “wall-wart” style power supply and uses the same cables as the batteries which power the system. The second port is the USB-C cable that can connect the system to the USB host port of a computer. Through this connection, a user can re-upload the controller program, download temperature data, or see debugging information. However, it is never strictly necessary for the operation of the Moonrat II.

### 2.1 Advantages

Our fully open-source portable incubator is designed to accelerate field work in the life sciences, providing robust and reliable microbial cultivation outside traditional laboratory settings. It offers customization of its design, focusing on portability, usability, and ruggedness.

Designed to be a standard Arduino UNO shield for the Arduino R4 Minima, the Moonrat II uses inexpensive, globally accessible components, enabling assembly in a wide range of international contexts at a cost (not counting labor) much less than regular desktop incubators. It consumes about 1.3 W of power under normal conditions, giving it a long battery life, and has a simple, 3-button interface that connects to a menu system.

This system, similar to [5], is far more portable than regular laboratory supplies.

Through harnessing the power of a portable incubator, long timelines for obtaining genuine scientific results will dramatically compress from weeks to mere days, enabling scientists and engineers to make crucial, in-the-field decisions.

The Moonrat II is designed for cost-effectiveness, portability and ease-of-use in rugged rural environments. Unlike traditional laboratory incubators, which usually cost more than $500 US dollars, our portable solution is designed to be affordable. Weighing about three pounds, it is much more portable than typical lab incubators (which are approximately 15–40 pounds), making our device easily transportable to remote and rugged environments. It operates on versatile 12-volt power sources, making it adaptable to various field conditions where 110VAC power may not be available.

The Moonrat II enables quick, on-site analysis of microbial contamination, which is crucial for water quality assessments in remote areas. This facilitates more rapid pathogen detection and monitoring during field studies, enhancing real-time decision making. The Moonrat II supports diverse biological experiments beyond standard assays, fostering innovation in field-based research methodologies. Finally, it is completely open source, and supports customization to better address a user’s specific needs.

These features and advantages aim to revolutionize field studies by reducing turnaround times for critical scientific analyses, ultimately improving public health and environmental monitoring efforts worldwide.

## 3 Design files summary

The Moonrat II project is a living, active project, which keeps all relevant design files in a GitHub repository [15]. Our recent release has been published on Zenodo for academic durability. Please see Table 1.

The firmware_moonratII directory contains a regression test plan and two subdirectories:

**Table 1:** Design files for the Moonrat II Portable Incubator.

Design filename	File type	Open source license	Location of the file
firmware_ Moonrat II	Directory with source files. A README.md describes the various source files	Firmware: Affero GPL 3.0	https://zenodo.org/records/20531233/preview/moonrat-1.0.4.zip?include_deleted=0#tree_item120

hardware_ moonratII	Directory holding a README describing the subdirectories to follow.	CERN Open Hardware Li- cence Version 2 - Strongly Reciprocal	https://zenodo.org/records/20531233/preview/moonrat-1.0.4.zip?include_deleted=0#tree_item145

pcb_controller_ moonratII	KiCad schematic and PCB files	CERN Open Hardware Licence Version 2 - Strongly Reciprocal	https://zenodo.org/records/20531233/preview/moonrat-1.0.4.zip?include_deleted=0#tree_item173

pcb_heater_ board_1. kicad_pro	KiCad schematic and PCB files	CERN Open Hardware Licence Version 2 - Strongly Reciprocal	https://zenodo.org/records/20531233/preview/moonrat-1.0.4.zip?include_deleted=0#tree_item295

enclosure_ moonratII	OpenSCAD and FreeCAD source files, STL and STEP files for the enclosure	CERN Open Hardware License Version 2 - Strongly Reciprocal	https://zenodo.org/records/20531233/preview/moonrat-1.0.4.zip?include_deleted=0#tree_item149

Manufacturing sub assemblies	Bill of Materials, Gerber files and description records of parts ordered for the builds	CERN Open Hardware Licence Version 2 - Strongly Reciprocal	https://zenodo.org/records/20531233/preview/moonrat-1.0.4.zip?include_deleted=0#tree_item145 Gerber files: https://zenodo.org/records/20531233/preview/moonrat-1.0.4.zip?include_deleted=0#tree_item209

Final assembly manufacturing and unit test documentation	Assembly instructions and notes	CERN Open Hardware License Version 2 - Strongly Reciprocal	https://zenodo.org/records/20531233/preview/moonrat-1.0.4.zip?include_deleted=0#tree_item150

• production: The main production files.
• factorytest: A simple factory test program for validation of hardware.

The hardware_moonratII directory contains the KiCad Schematic and PCB design files, KiCad library files, a Manufacturing README.md for the printed circuit assembly, and other manufacturing documentation in four subdirectories:

• hardware_moonratII/pcb_heater_board_1.kicad_pro: PCB design files for a small assembly placed inside the incubation chamber. It connects via a ribbon cable to the processor and provides mounting points for the digital thermometer and heating cloth.
• hardware_moonratII/enclosure_moonratII: The FreeCAD source files and generated .STL files for 3D printing the plastic controller enclosure.
• hardware_moonratII/culture_holders: Petrifilm holder .STL files for the team-designed Petrifilm holders.
• hardware_moonratII/pcb_controller_moonratII: Gerbers, BoMs, and placement files used to order from JLCPCB, a PCB assembly manufacturing company.

### 3.1 Bill of materials

• KiCad generated spreadsheets pcb_controller_BOM.csv and pcb_heaterboard_1_BOM.csv include the parts list and usage instructions.
• JLCPCB built the raw PCBs, performed surface-mount device (SMD) assembly, and completed some through-hole assembly. Remaining through-hole assembly was completed by Public Invention volunteers.
• The first BOM below covers external purchases only, and the second BOM covers the components on the PCB assemblies (see Table 2).

These parts cost a total of $209.22.

**Table 2:** Bill of Materials (BOM) for the Moonrat II.

Designator	Component	Number	Cost per unit - currency	Total cost - currency	Source of materials	Material type
Thermos™	Big Boss Thermos Food Jar	1	69.99	69.99	thermos	Stainless steel

Battery	Rechargable 12 V 6 Ah	1	26.39	26.39	Amazon	Lithium

Battery charger	9–12 V wall power supply	1	8.95	8.95	Adafruit	Composite

Digital thermometer	Digital Thermometer	1	10.95	10.95	Sparkfun	Electronics

Heating pad	Heating Pad	2	5.95	11.90	Adafruit	Plastic

Ribbon cable	6${}^{\prime \prime }$ 10 pin ribbon cable	1	2.00	2.00	Adafruit	Plastic

Connectors	Conn 02x05 Odd Even	2	…	…	…	…

OLED screen	HS96L03W2C03 0.96 Oled	1	18.00	18.00	aliexpress	Semiconductor

Headers	PZ254V-11-04P	2	0.02	0.02	JLCPCB	Plastic

Arduino	Arduino R4	1	15.00	15.00	Arduino Store	electronics

Arduino shield header set	Arduino R4	Qty: 2, 1 × 8, Qty: 1, 1 × 10, Qty: 1, 1 × 6	2.50	2.50	Sparkfun (cheaper if not bought as a kit)	Plastic

Cylinder	General Purpose Aluminum Tubing, 3-1/2${}^{\prime \prime }$ OD, 0.049${}^{\prime \prime }$ Wall Thickness, 1 Foot Long	1	41.80	41.80	McMaster-Carr	Metals

Ends	Multipurpose 6061 Aluminum Sheet, 0.050${}^{\prime \prime }$ Thick, 6${}^{\prime \prime }$ x 6${}^{\prime \prime }$	2	5.11	10.22	McMaster-Carr	Metals

(Table 3) shows the parts for the main controller printed wiring assembly. The small components are provided by the PCB manufacturing house and are not priced separately.

The populated PCB has a unit price of $23.92 when 5 are ordered. These parts in total (PCB and external parts) cost $224.53 at the time of this writing.

**Table 3:** Printed circuit board small components.

Designator	Component	Number	Cost per unit - currency	Total cost - currency	Source of materials	Material type
D1	Schottky2A C14996	1	Part of PCB price	Part of PCB price	JLCPCB	Small PCB component

BZ1	Buzzer_D12 mm_ H9.6 mm_LS7.6 mm C360615	1	Part of PCB price	Part of PCB price	JLCPCB	Small PCB component

J5, J10	Conn_02x05_ Odd_Even C5665	2	Part of PCB price	Part of PCB price	JLCPCB	Small PCB component

J2	HS96L03W2C03_0.96_ Oled C5248080	1	Part of PCB price	Part of PCB price	JLCPCB	Small PCB component

U1	TMP36GRTZ-REEL7 C129489	1	Part of PCB price	Part of PCB price	JLCPCB	Small PCB component

J11	Screw_Terminal_ 01x02 C474920	1	Part of PCB price	Part of PCB price	JLCPCB	Small PCB component

R2, R3, R4, R5, R6	C269704	6	Part of PCB price	Part of PCB price	JLCPCB	Small PCB component

Q2	BSS138	C400505	Part of PCB price	Part of PCB price	JLCPCB	Small PCB component

Q1	IRF520N	C2598	Part of PCB price	Part of PCB price	JLCPCB	Small PCB component

MF1, MF2, MF3 CAPTACTILEROUNDIVORY	C231316	3	Part of PCB price	Part of PCB price	JLCPCB	Small PCB component

MF12	straight pins C2691448	1	Part of PCB price	Part of PCB price	JLCPCB	Small PCB component

J6, J9	Screw_Terminal_01x06 C474924	2	Part of PCB price	Part of PCB price	JLCPCB	Small PCB component

2R1, R7	1R C269434	3	Part of PCB price	Part of PCB price	JLCPCB	Small PCB component

F1	Polyfuse6A	C883162	Part of PCB price	Part of PCB price	JLCPCB	Small PCB component

MF4, MF5, MF6, MF8	round nut C3020130	4	Part of PCB price	Part of PCB price	JLCPCB	Small PCB component

J1	Barrel_Jack_Switch_SMT	1	Part of PCB price	Part of PCB price	JLCPCB	Small PCB component

SW1, SW2, SW3	6 mm2Pin C87037	3	Part of PCB price	Part of PCB price	JLCPCB	Small PCB component

## 4 Build instructions

Partial assembly of the Moonrat II consists in Printed Wiring Assembly (PWA) fabrication. Most PWA components are from JLCPCB. Public Invention added the components that are not available at JLCPCB.

### 4.1 Tools required

• **Drill press**
• **Metal bits, approximately 3/8** ${}^{\prime \prime }$
• **Soldering iron, solder, and flux**
• **Hot melt glue gun**
• **#1 Phillips screwdriver**
• **Round metal file**
• **Hack saw**
• **Heavy tin snips**

### 4.2 Ordering PWA subassembly

The final controller is depicted in Fig. 1(a), and the final heater is depicted in Fig. 1(b).

Fig. 1The PWA and PWA heater assembly, both received from JLCPCB.Fig. 1

**(a):**
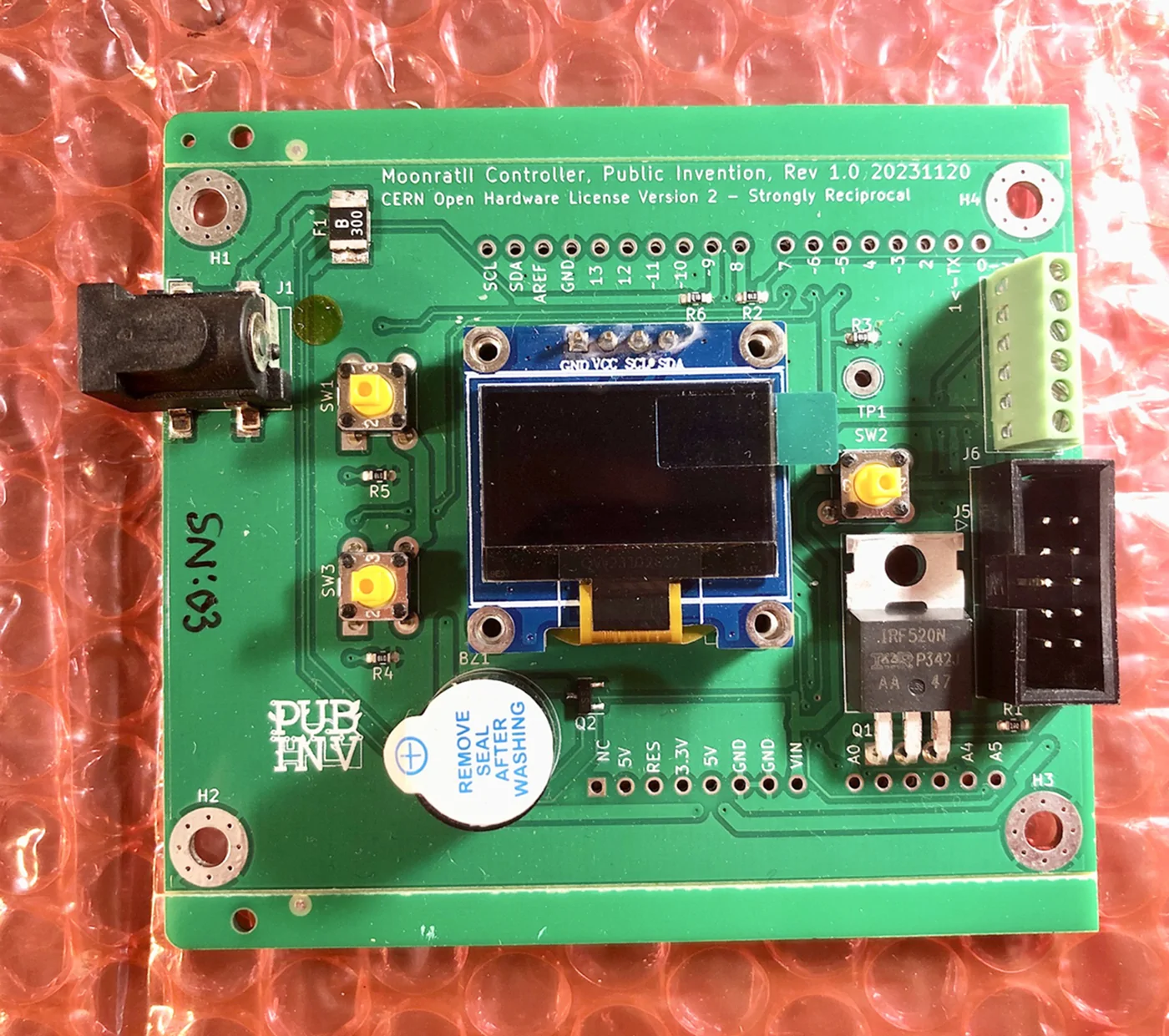
.

**(b):**
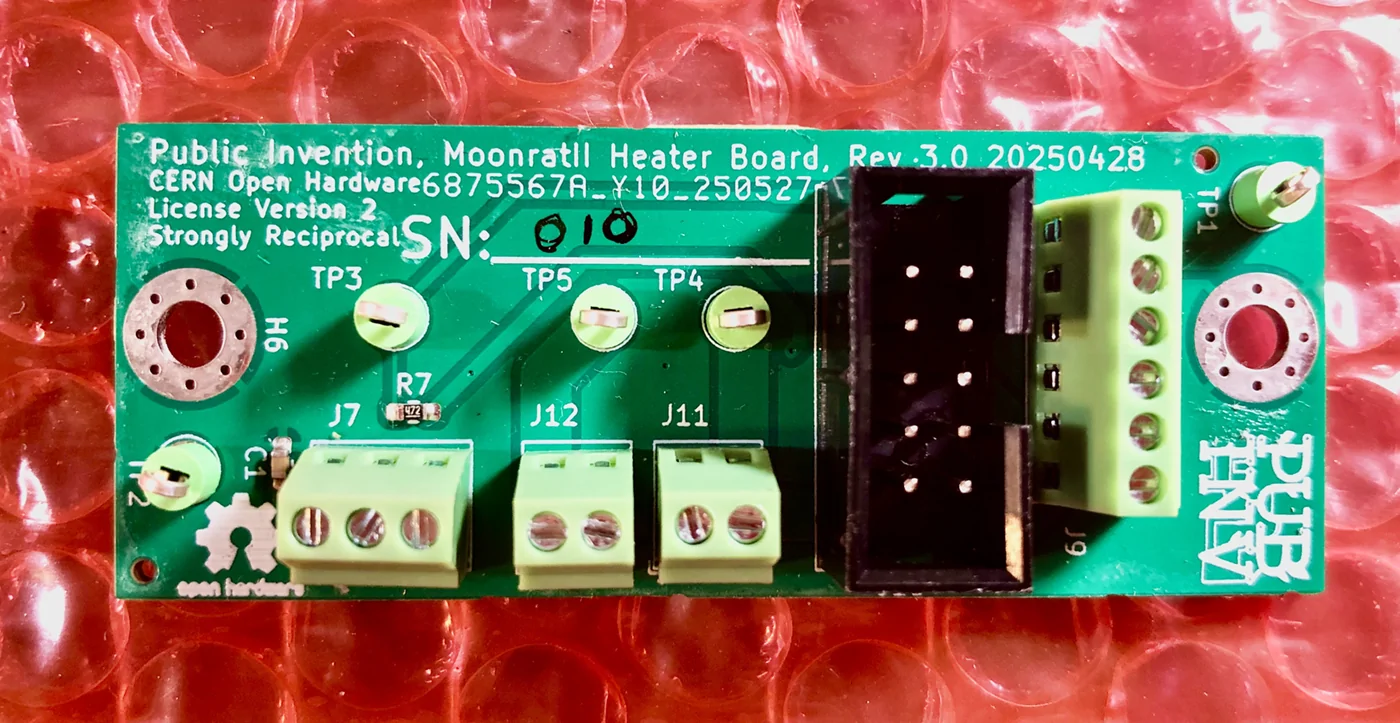
.

### 4.3 3D printing

We 3D printed a keycap for each of the three buttons using the file:

• hardware_moonratII/enclosure_moonratII/Parts/switch_cap_b32-2010.stl

### 4.4 Modifying the incubation chamber: Detailed assembly instructions (Drilling/Insulating )

The incubation chamber enclosure was 3D-printed using a standard home printer. Print one enclosure per assembly using the following files:

• https://zenodo.org/records/20531233/preview/moonrat-1.0.4.zip?include_deleted=0#tree_item163 **(then look for base.FCStd)**
• https://zenodo.org/records/20531233/preview/moonrat-1.0.4.zip?include_deleted=0#tree_item163 **(then look for cover.FCStd)**

1. 3D print all of the enclosure parts. We recommend ABS or Nylon as a durable material.
2. Obtain a Big Boss Thermos™ vacuum flask as depicted in Fig. 2.
3. Drill a hole in the container lid, as shown in Fig. 3, to allow the ribbon cable to connect to the heater board. This can be accomplished by drilling multiple small holes and enlarging them with a file, or by drilling a single, larger hole directly.
4. Insert the ribbon cable into the heater board as per Fig. 4.
5. Use hot melt glue to fill the hole with the ribbon cable to prevent wear on the ribbon cable and insulate the chamber.
6. Then attach the heater board to the inside of the lid as shown in Fig. 5 with hot melt glue.
7. Screw the heater board into the cage (see Fig. 6).
8. Mount the cage inside the incubation container with hot melt (as per Fig. 7).

**Fig. 2:**
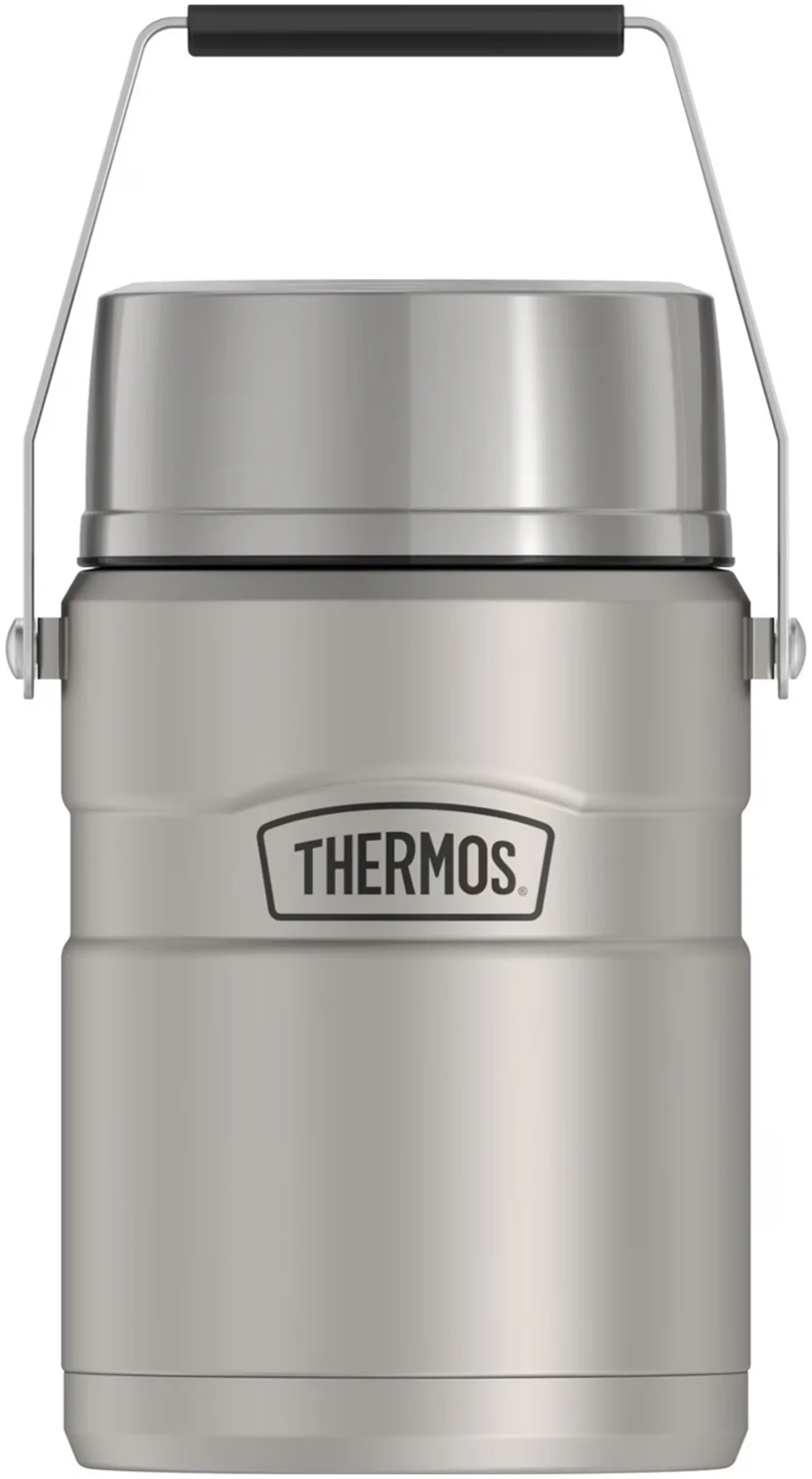
Vacuum flask container to be modified to build the Moonrat II.

**Fig. 3:**
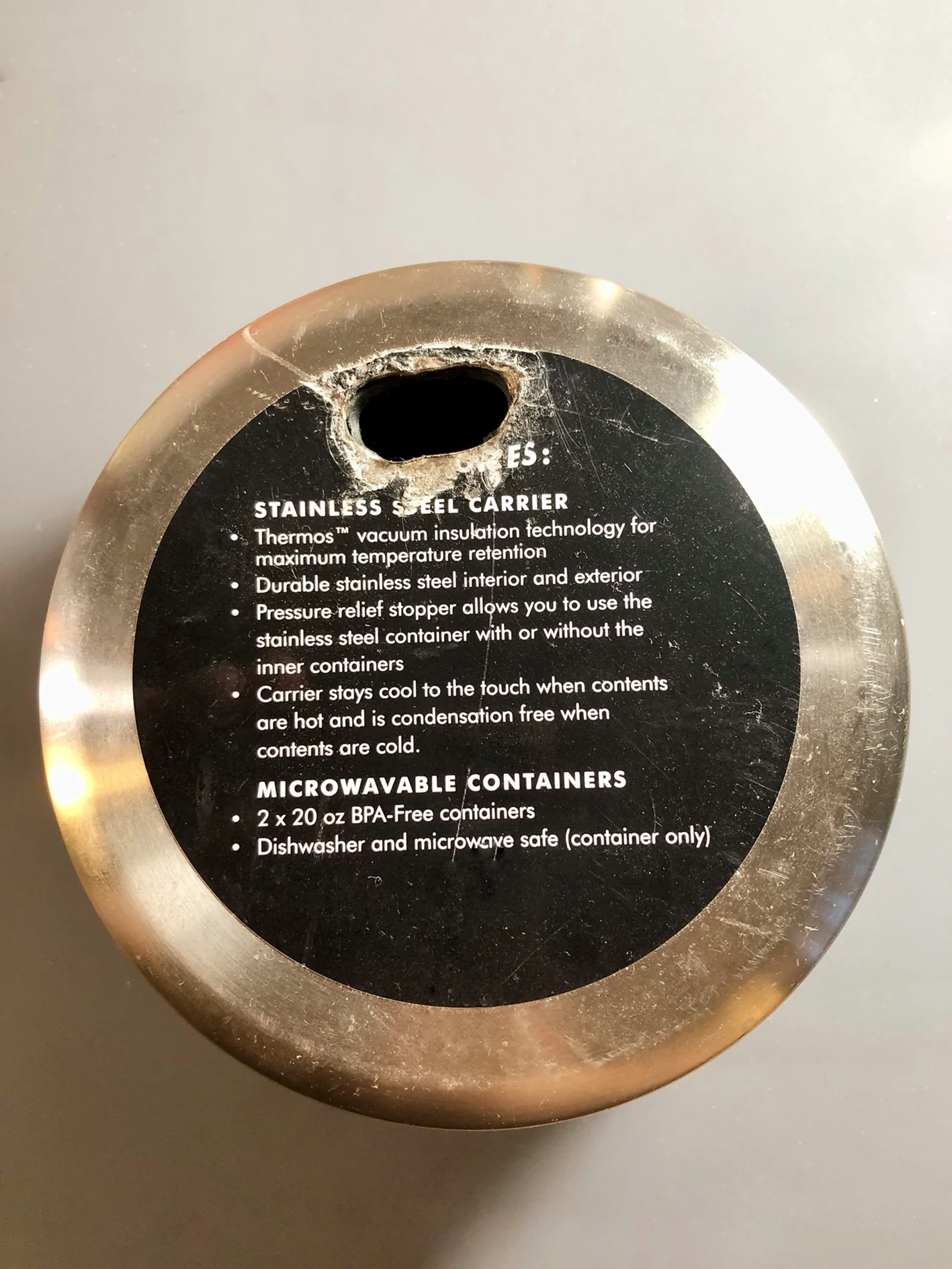
Drilled hole in lid of vacuum flask for wires.

**Fig. 4:**
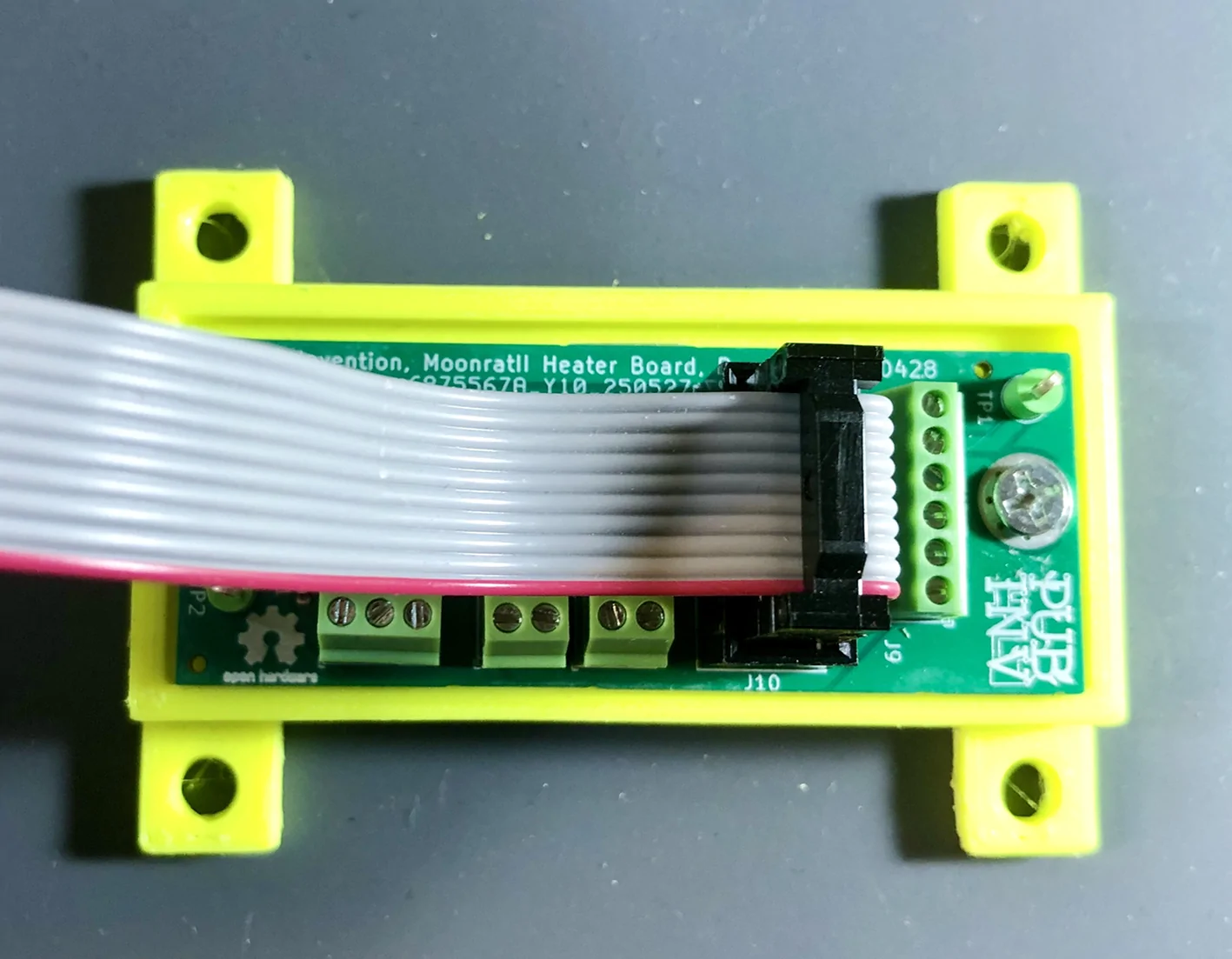
Ribbon cable inserted into heater board.

**Fig. 5:**
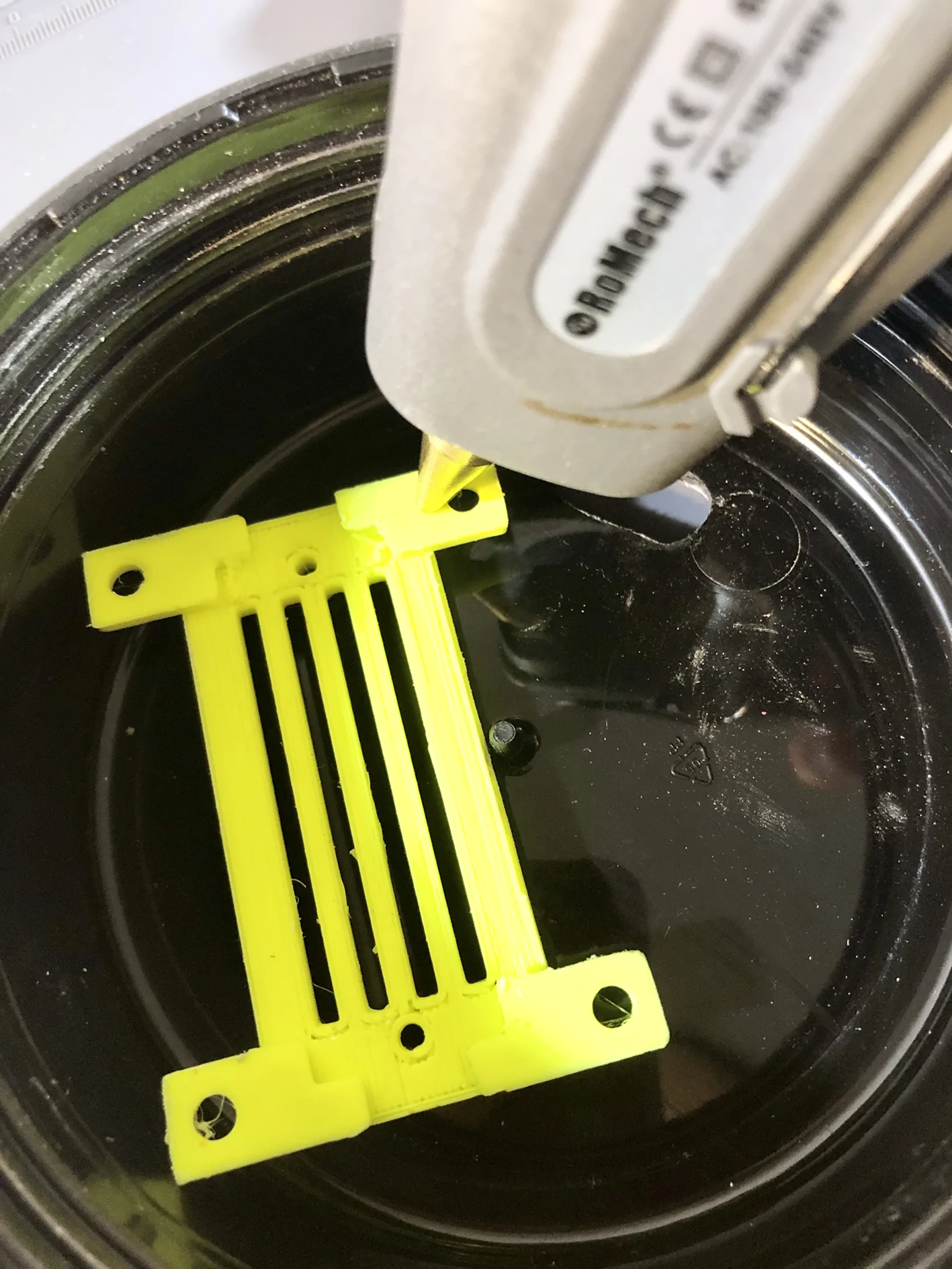
Heater board being attached to the underside of the lid.

**Fig. 6:**
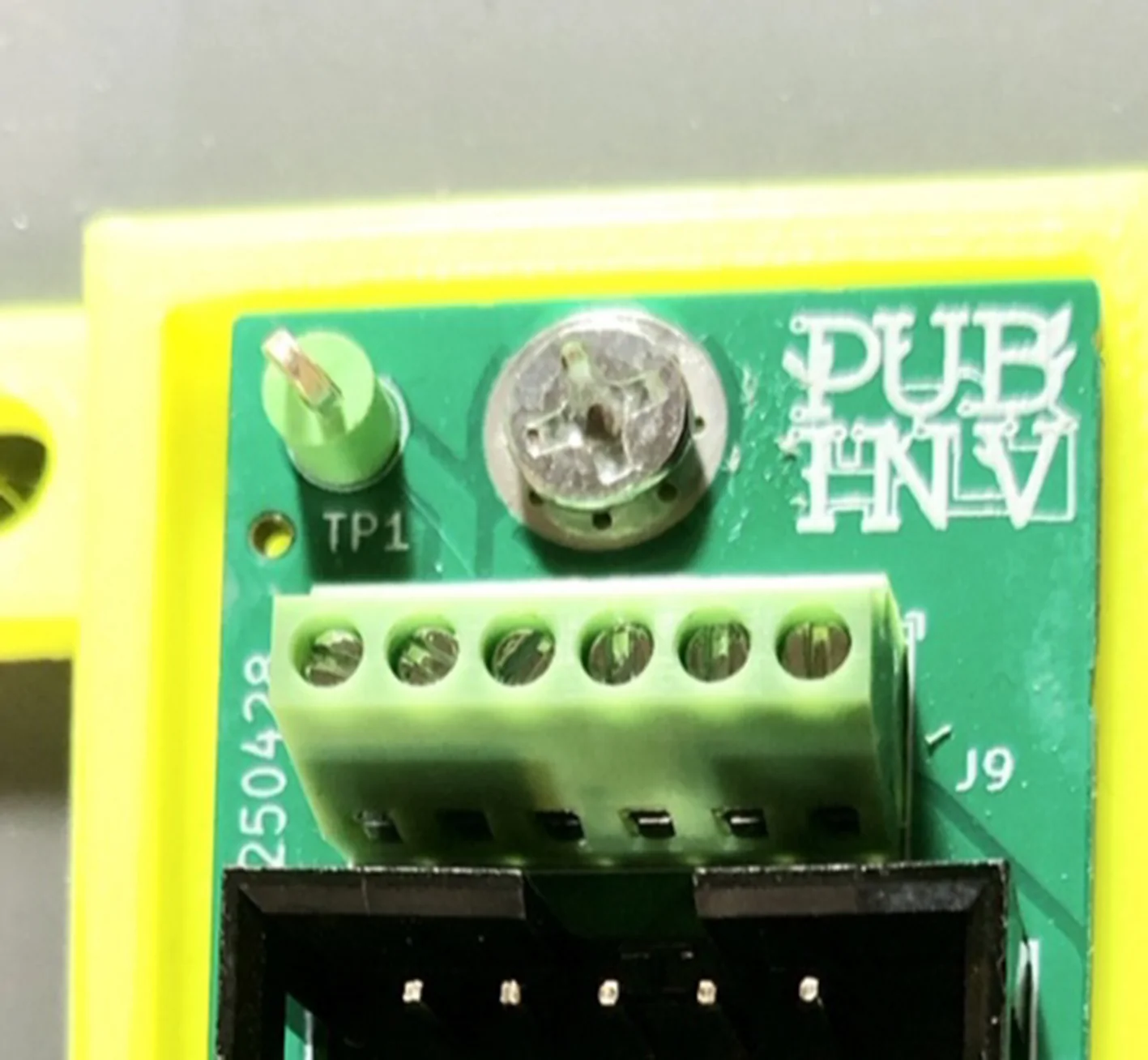
Close up of the heater board.

**Fig. 7:**
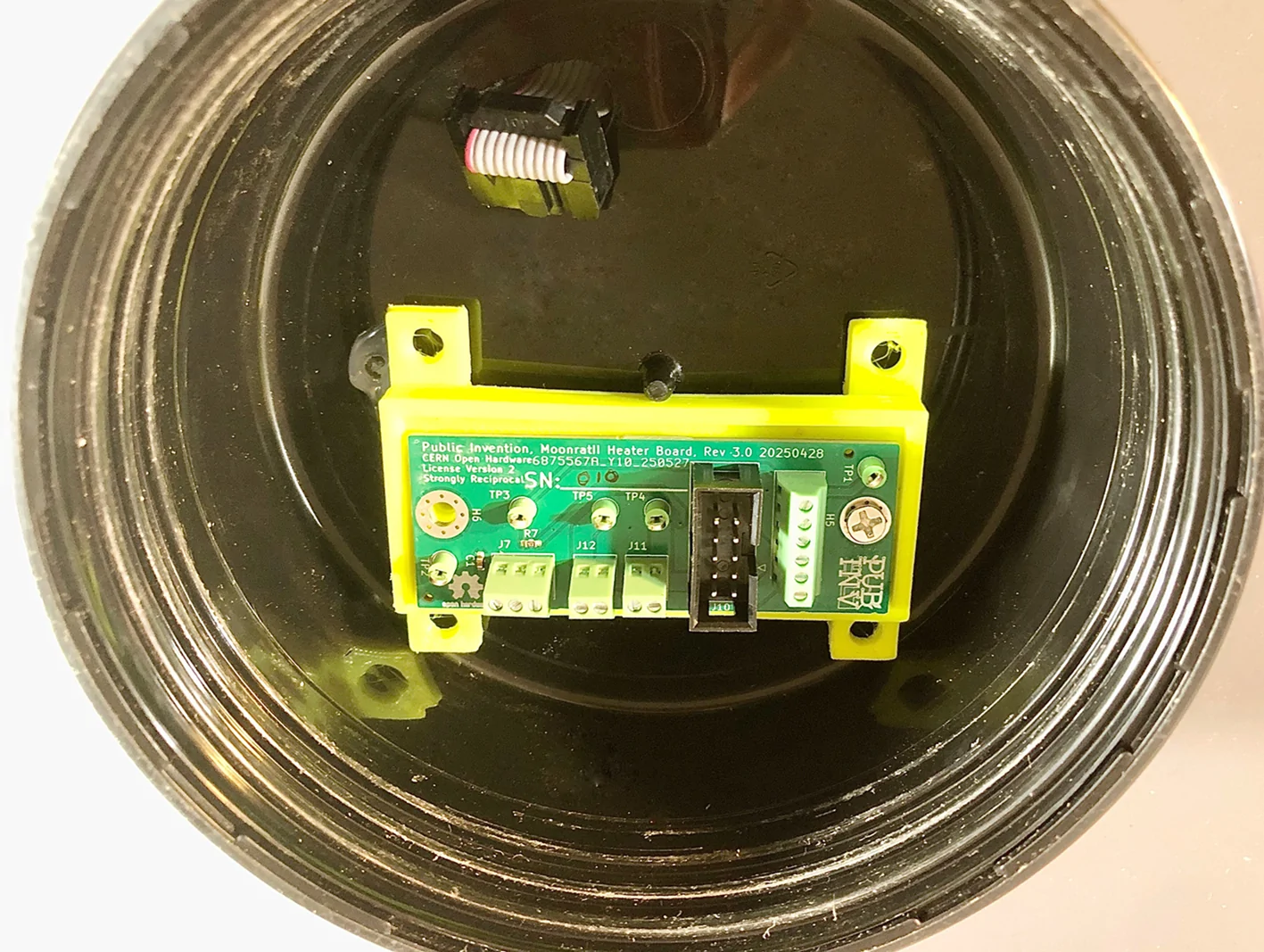
Heater board cage glued to underside of lid.

### 4.5 Assembly of controller

1. Solder four (1–10 pin, 2–8 pin, 1–6 pin) (Fig. 8) headers to the PCB.
2. Add keycaps to the 3 switches as shown in Fig. 9.
3. Thread the ribbon cable through the drill-pressed hole and then insulate the hole with hot melt glue, as shown in Fig. 10.
4. Attach UNO R4 Minima to the PWA to create a controller assembly (Fig. 11).
5. Place the controller assembly into the top and bottom enclosure pieces (Fig. 12).
6. Screw together the top and bottom controller enclosure pieces.
7. Attach the controller assembly to the top exterior of the container as in Fig. 13.

**Fig. 8:**
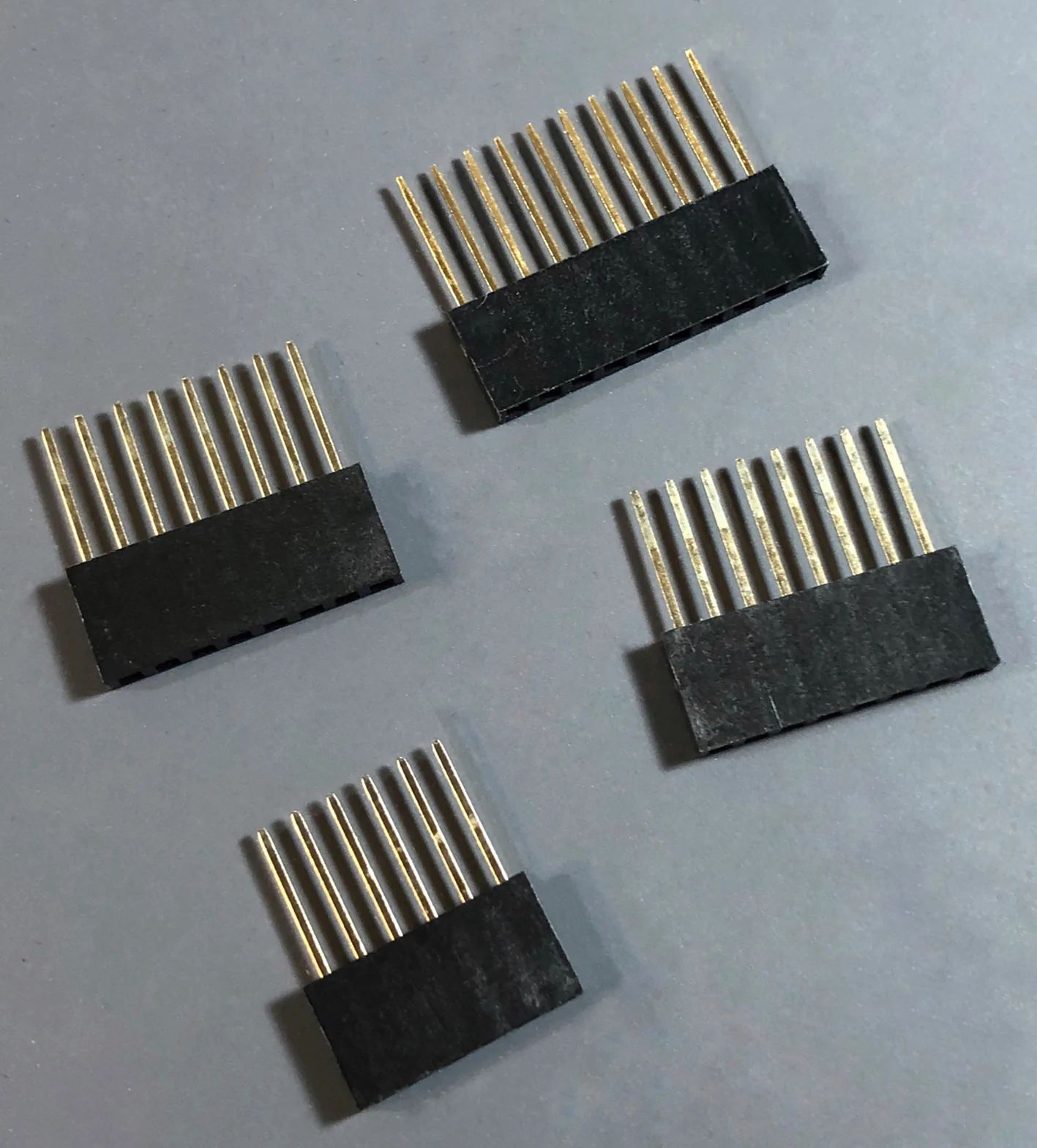
Female 0.10 pitch female headers.

**Fig. 9:**
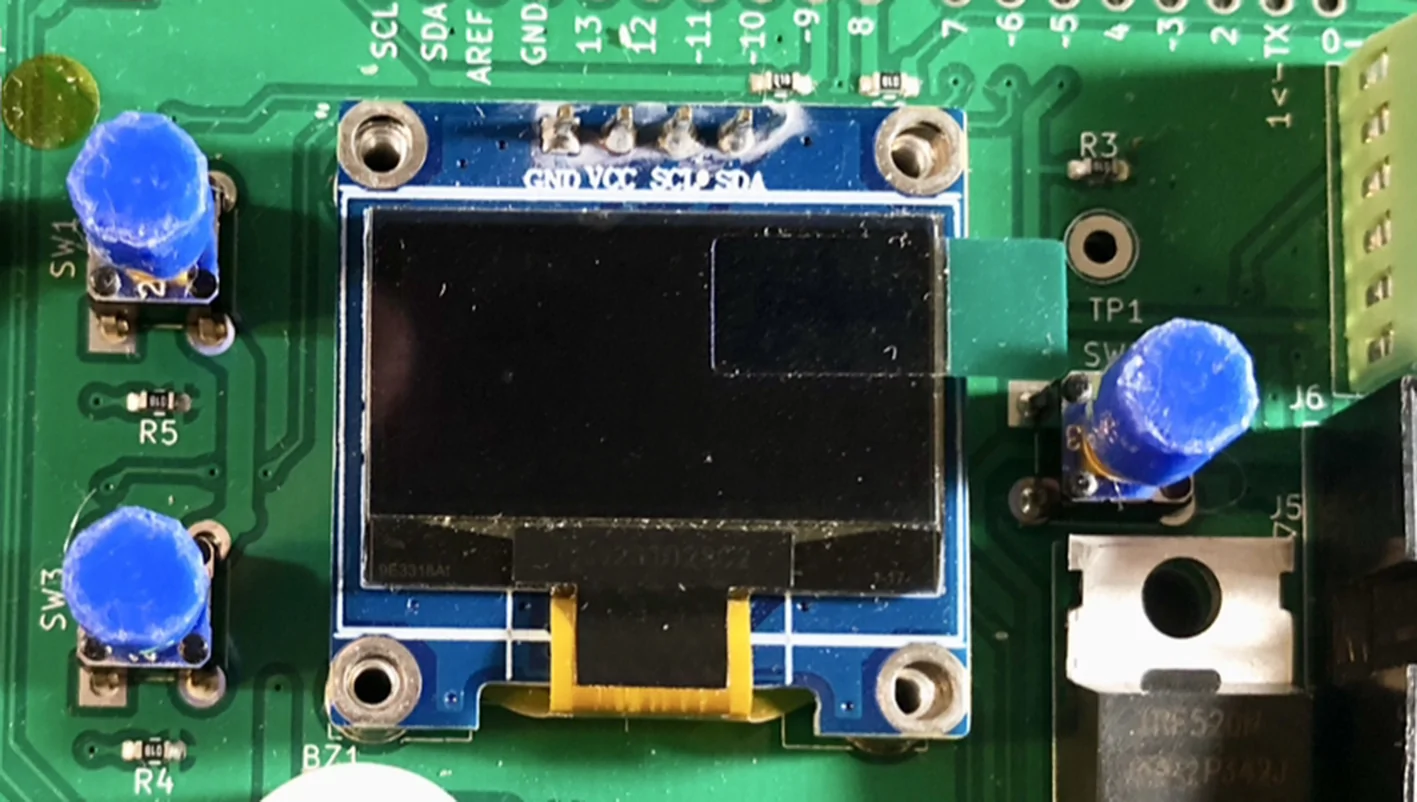
3D printed keycaps pressed onto buttons.

**Fig. 10:**
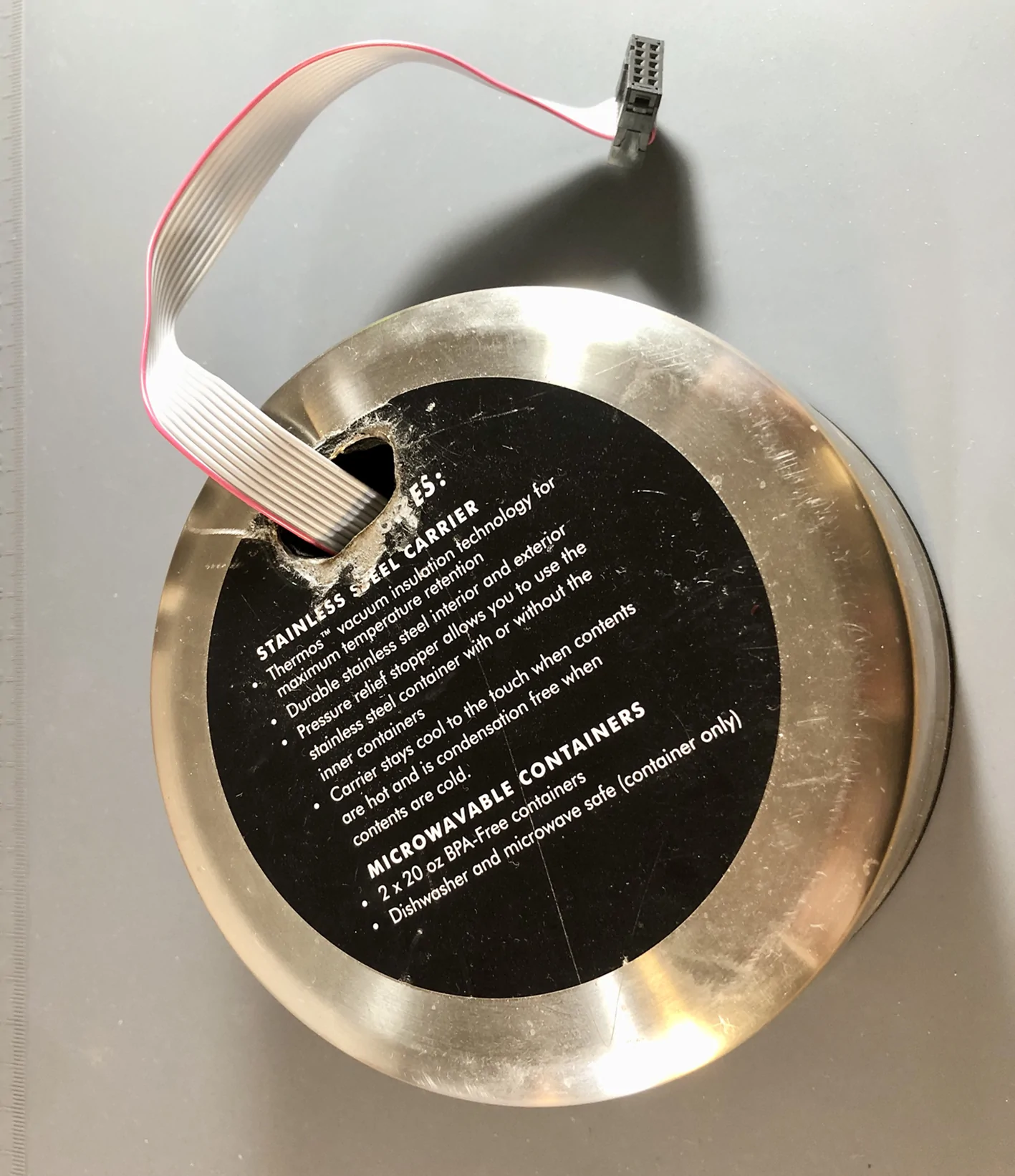
Ribbon threaded through hole in lid.

**Fig. 11:**
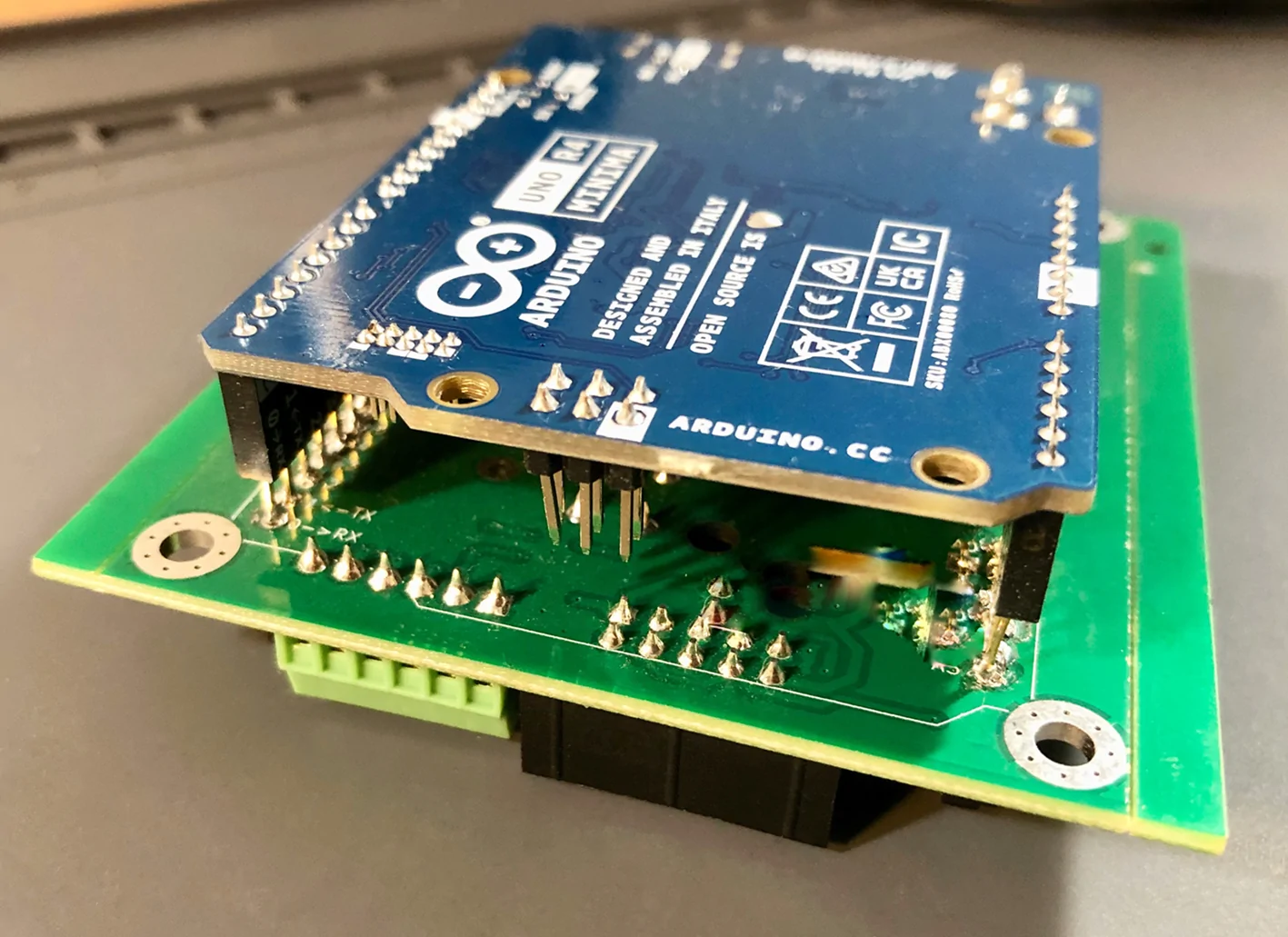
Arduino R4 plugged into controller board “shield”.

**Fig. 12:**
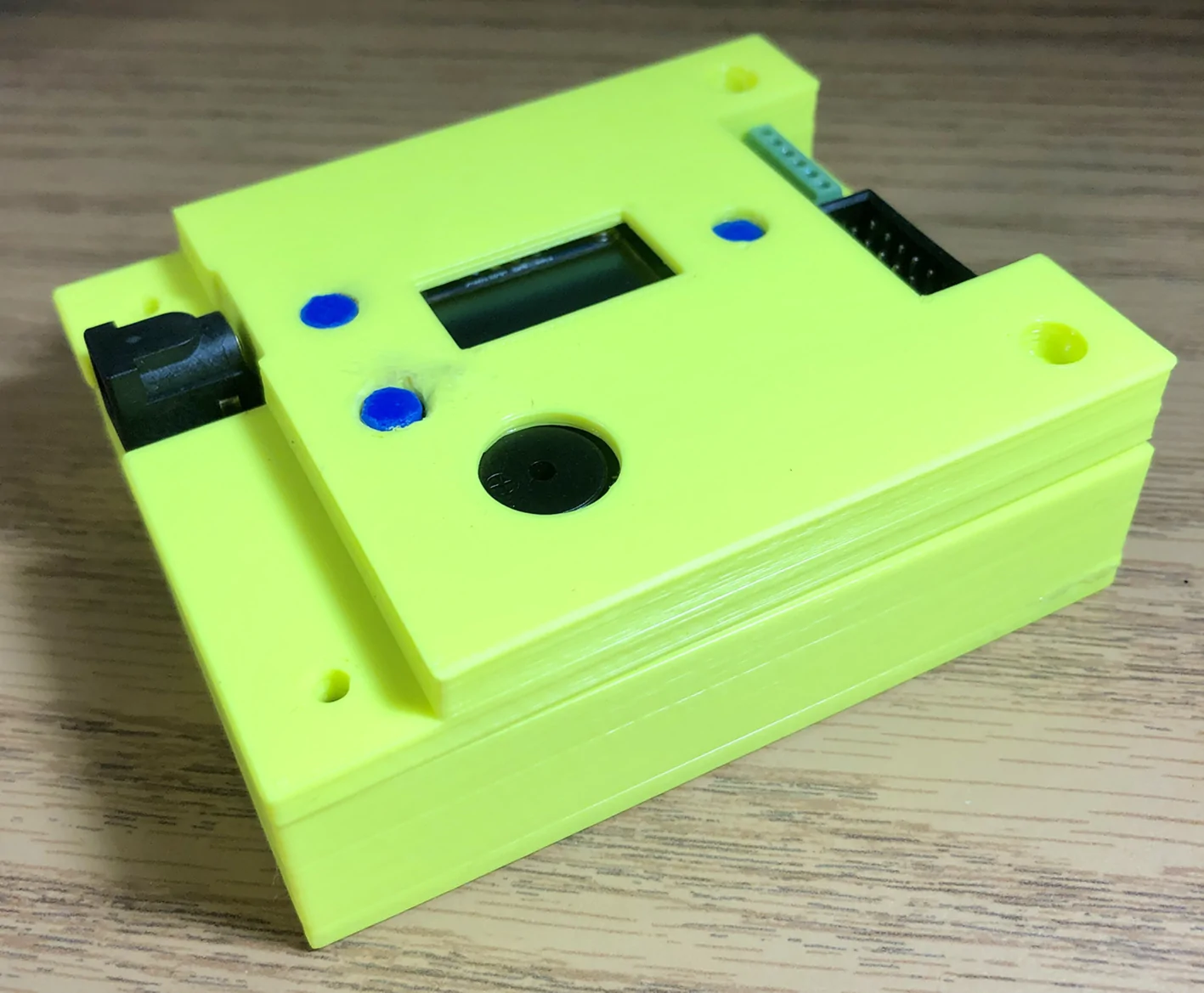
Assembled Moonrat II controller enclosure loaded with controller board.

**Fig. 13:**
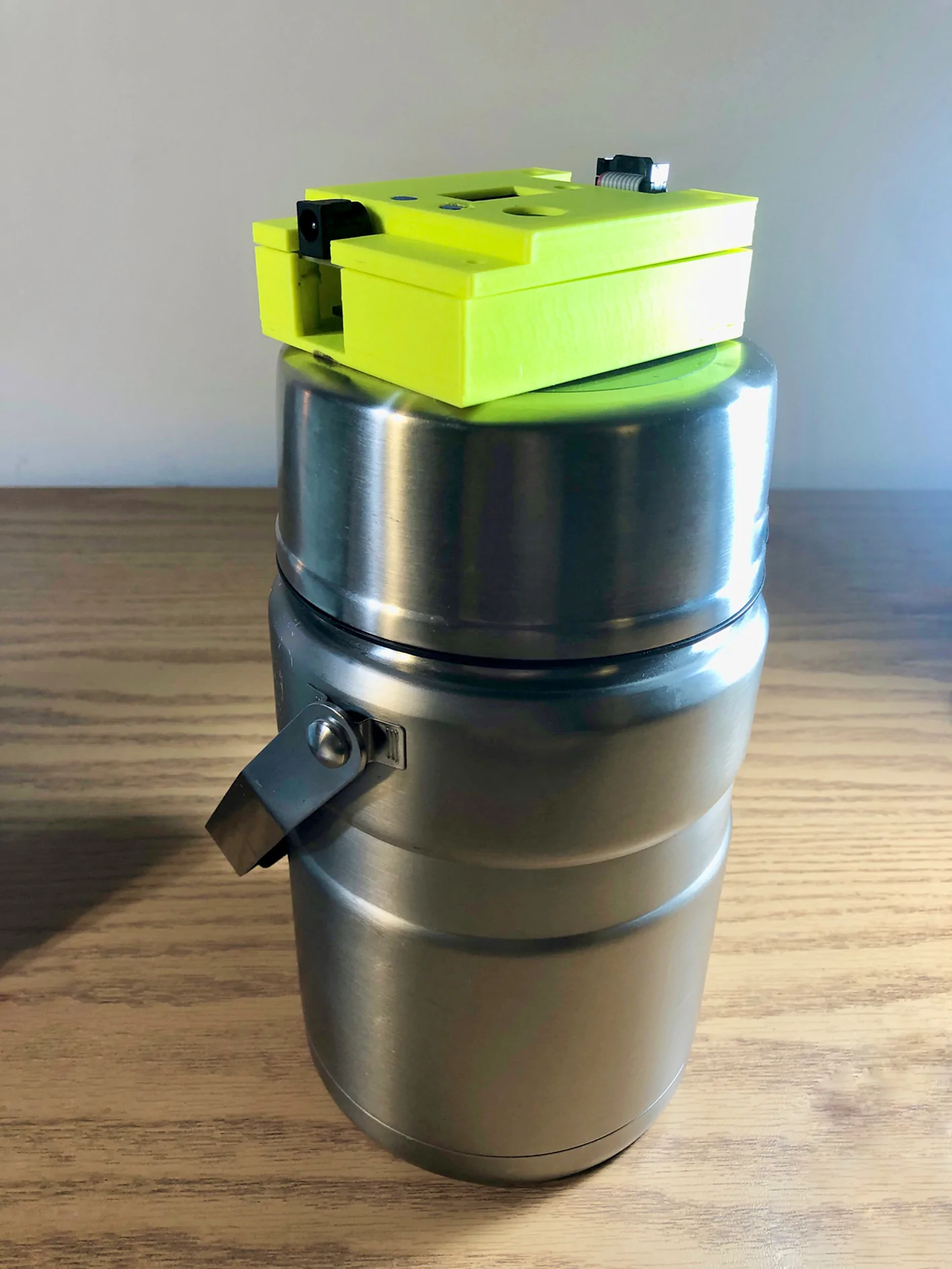
Fully assembled Moonrat II controller and incubator.

### 4.6 Assembly of heater board

1. Screw clamp the digital thermometer (Fig. 14).
2. Screw clamp the heating cloth. The two heating cloths should be wired to each other in a series, producing a single load of 12.7 Ω.
3. Plug the ribbon cable into the heater board socket as shown in Fig. 15.
4. Insert the heater board into its tray to create a heater board assembly (Fig. 16).
5. Make the interior aluminum cylinder, depicted in Fig. 20:
  • Cut the aluminum tube with a hacksaw to a 160 mm length, as depicted in Fig. 18.
  • Cut out two aluminum discs by tracing the 3.5${}^{\prime \prime }$ OD tube onto the sheet metal and then using the tin snips to cut out discs.
  • Smooth the discs with the file.
  • Tape the bottom onto the cylinder with duct tape as shown in Fig. 19.
  • Cut a “u”-shaped notch in the top disc with the snips and the file, as shown in Fig. 17.
6. Mount the digital thermometer inside the heating chamber (Fig. 14).
7. Solder the heating cloths in a series with about 6${}^{\prime \prime }$ between them, and with 8${}^{\prime \prime }$ leads.
8. Wrap the heating cloth around the cylinder. Be sure that about 20% of each cloth is taped in contact with the top and bottom. The heating cloth must be in contact with the top and the bottom to make the axial temperature difference small. This is depicted in Figs. 21(b), 21(c), and 21(a).

The notched top should be hinged with duct tape as shown in Fig. 21(c).

**Fig. 14:**
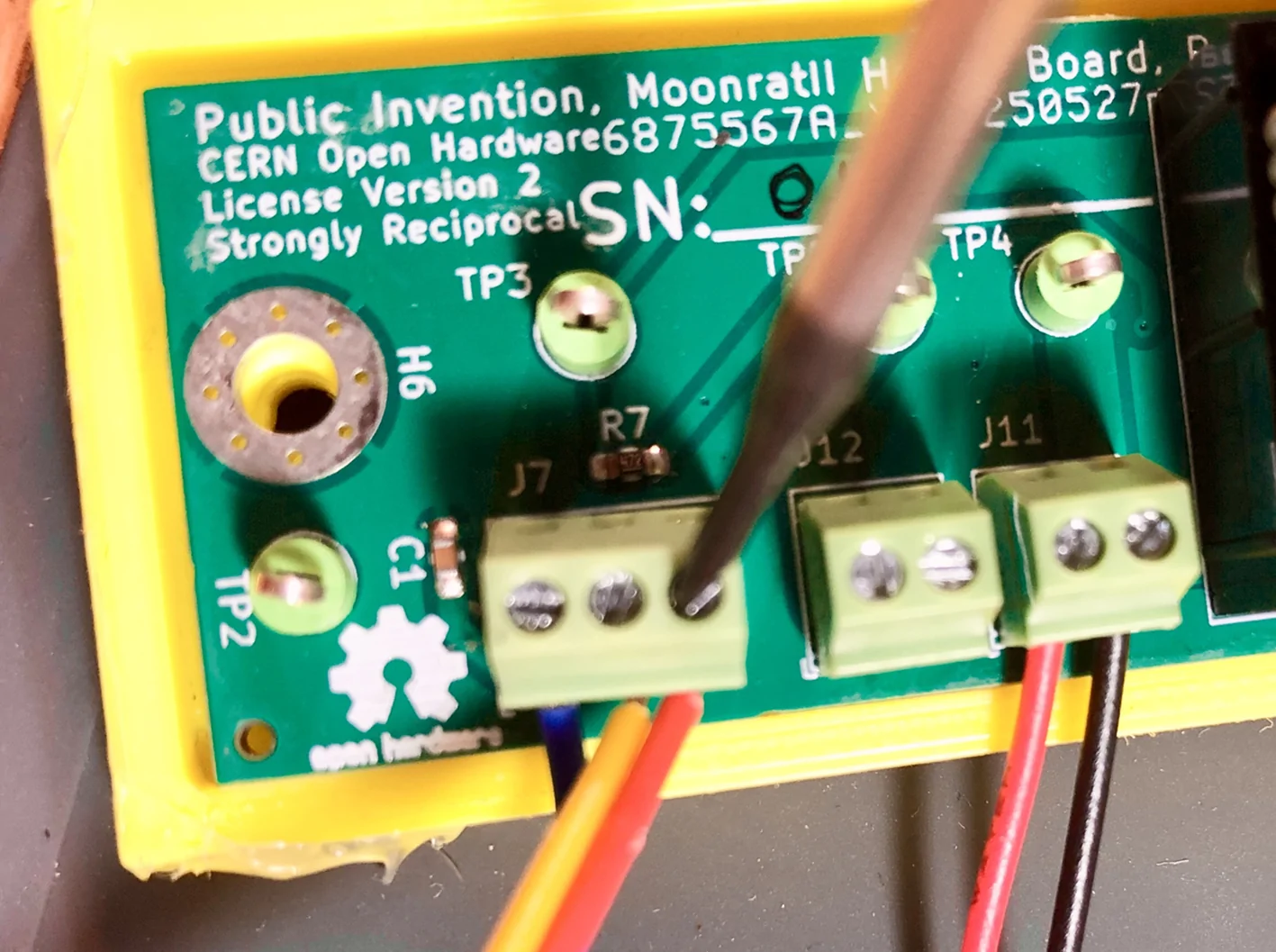
Digital thermometer being attached.

**Fig. 15:**
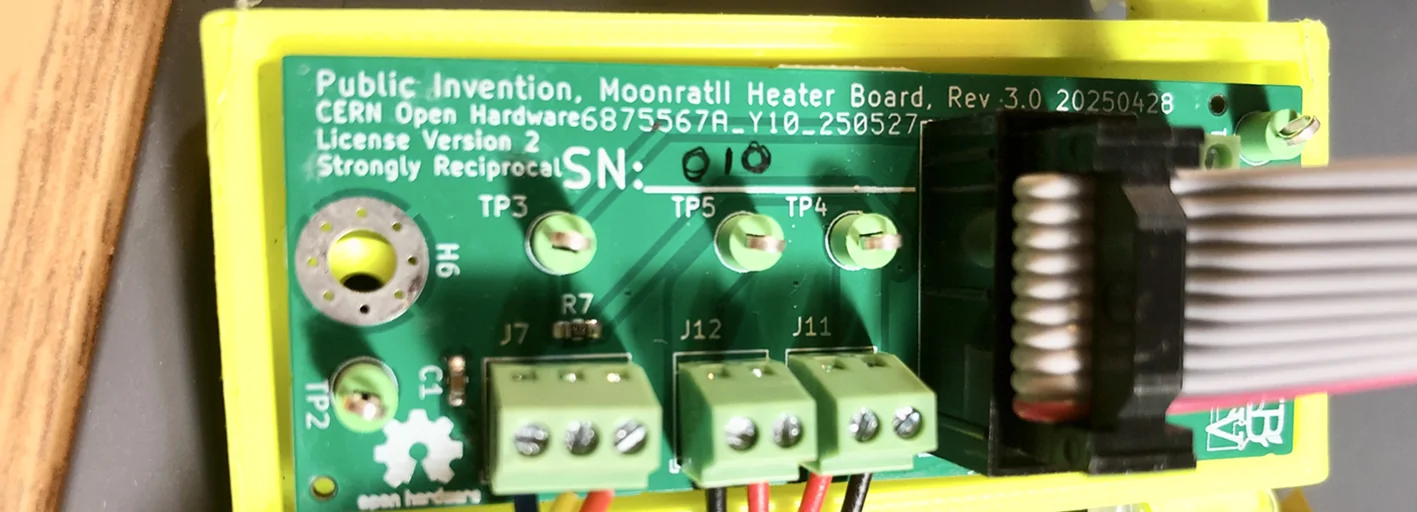
Ribbon cable attached to heaterboard.

**Fig. 16:**
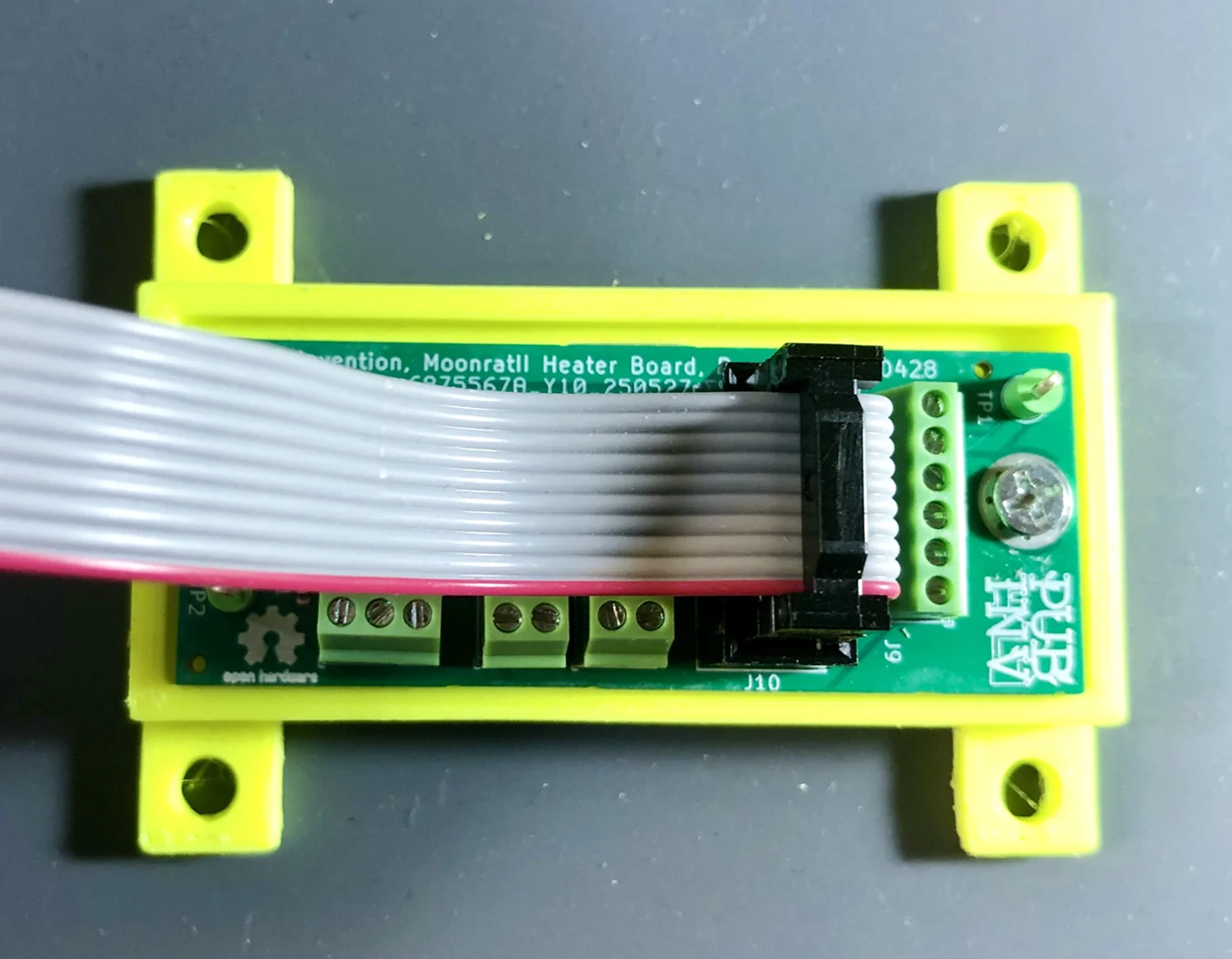
Heater board attached to holding tray.

**Fig. 17:**
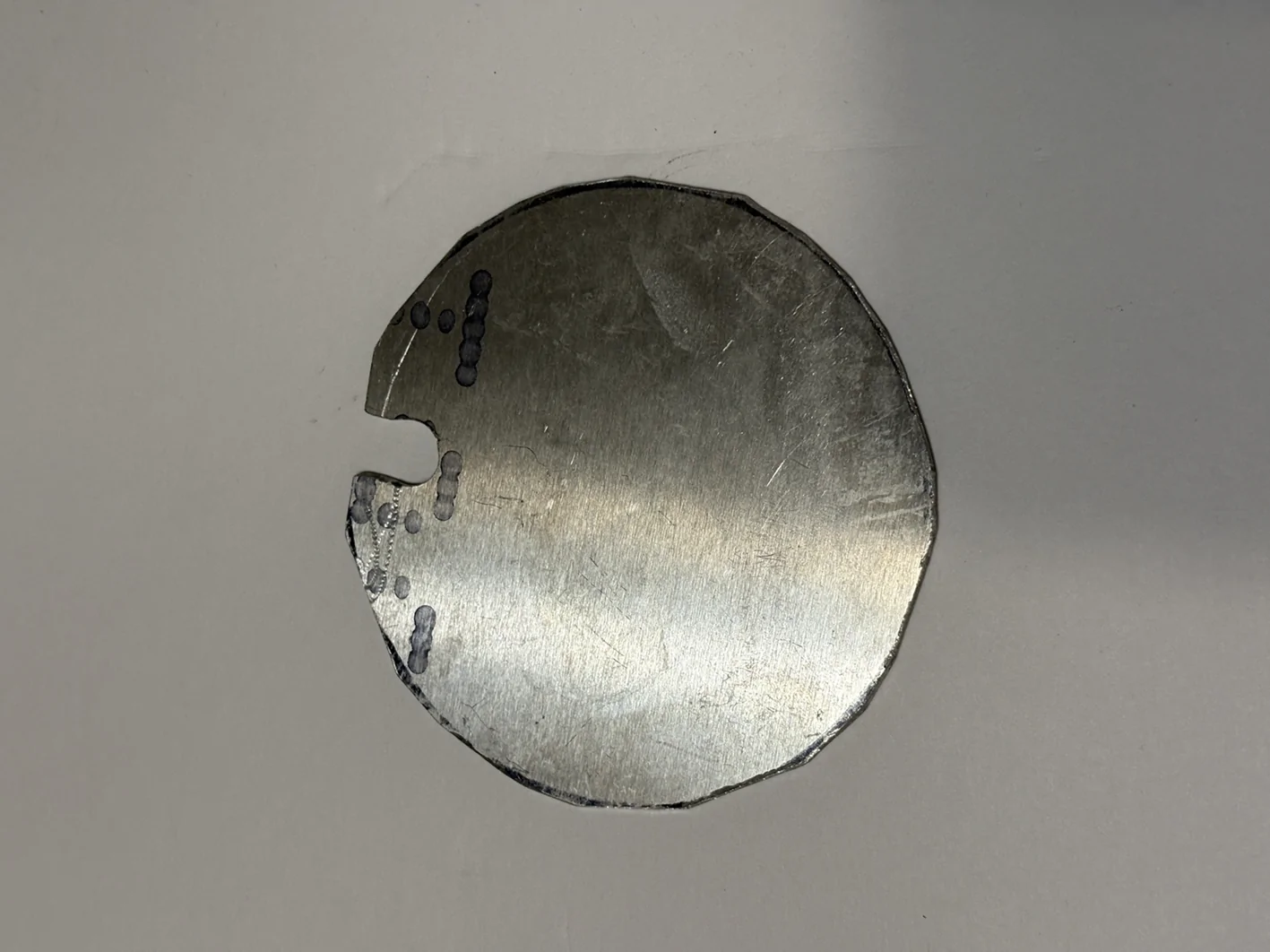
End cap with a U-notch cut out for temperature probe.

**Fig. 18:**
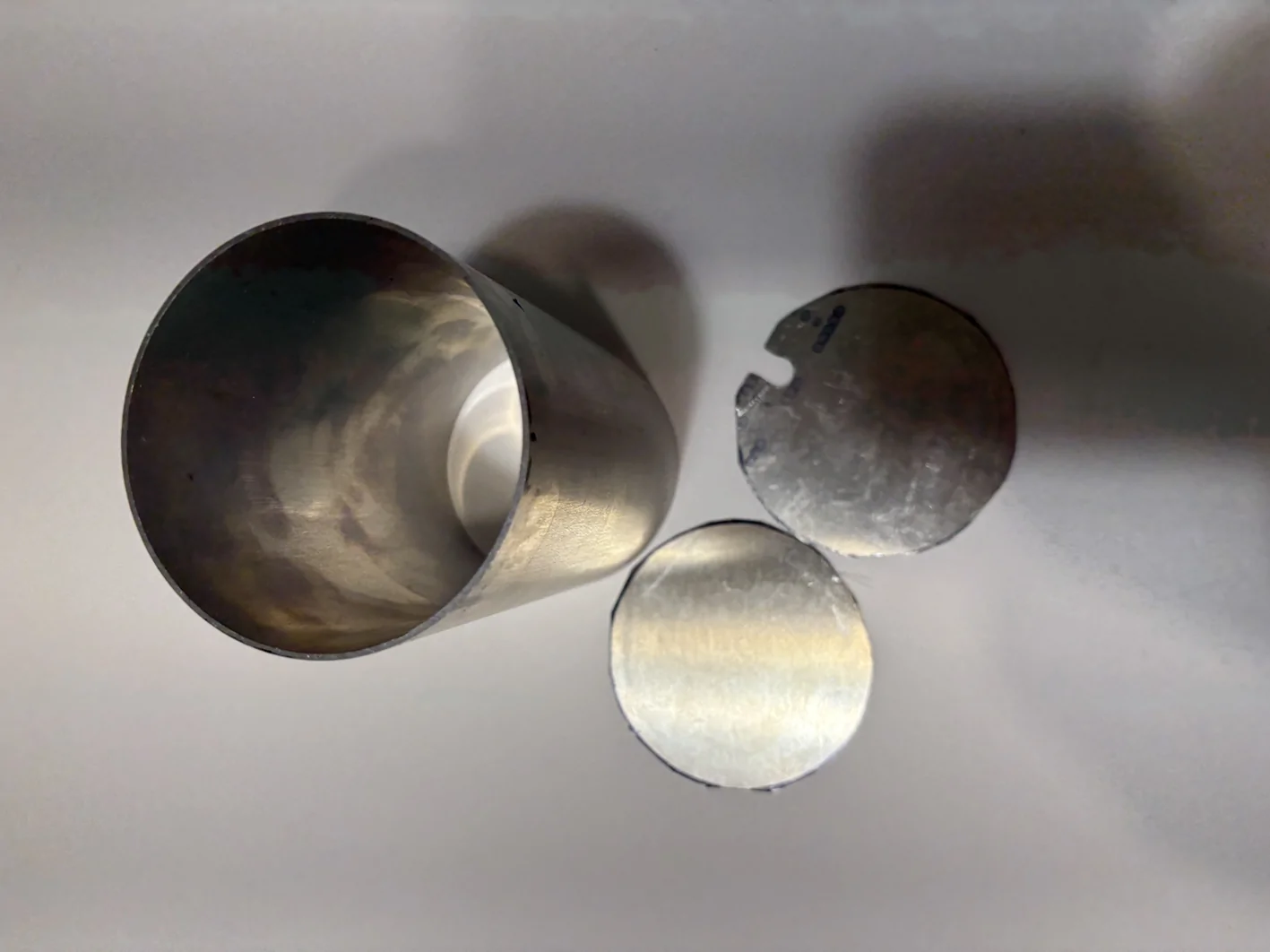
The aluminum cylinder and round end caps.

**Fig. 19:**
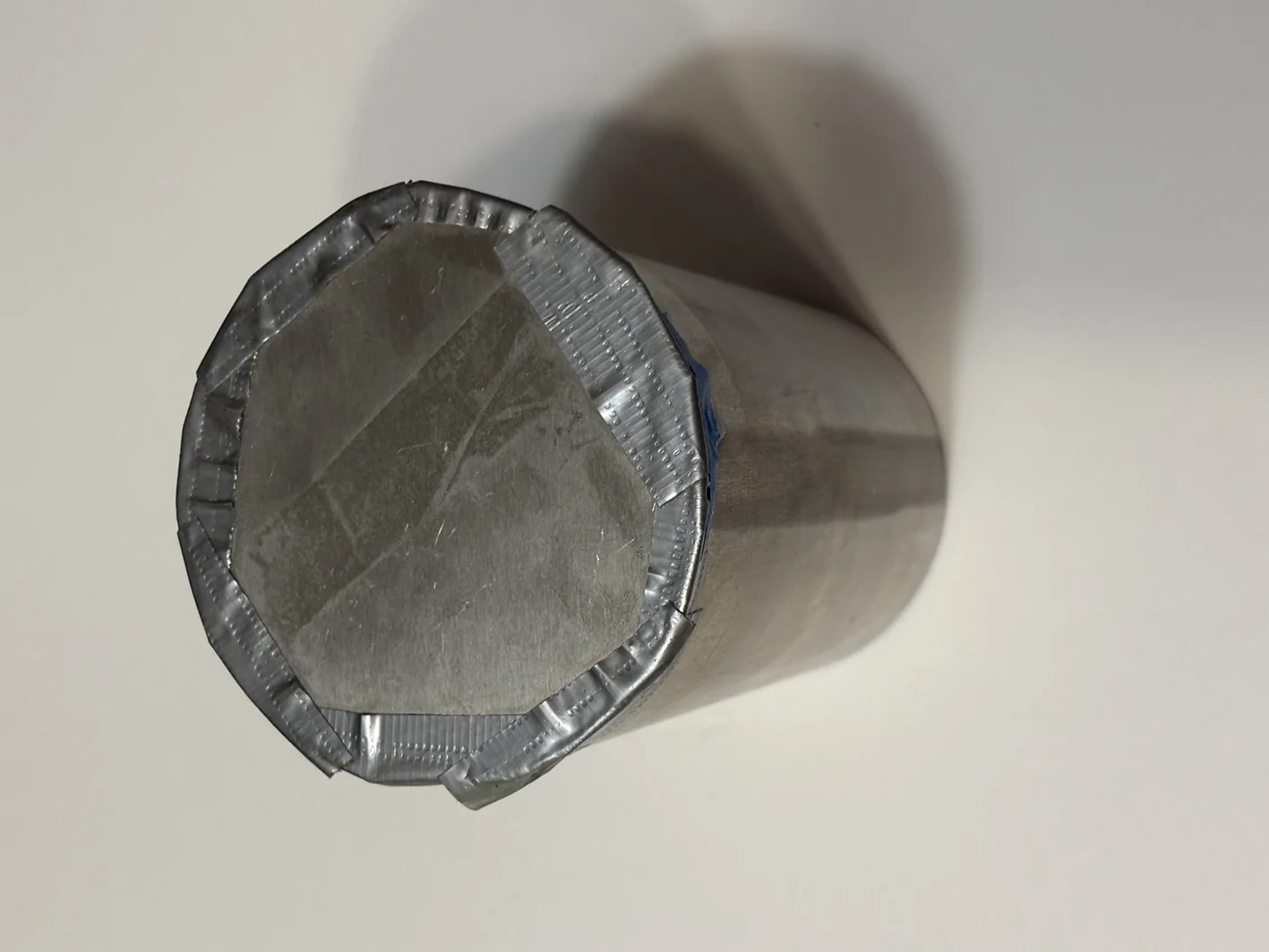
The aluminum cylinder with bottom end cap taped.

**Fig. 20:**
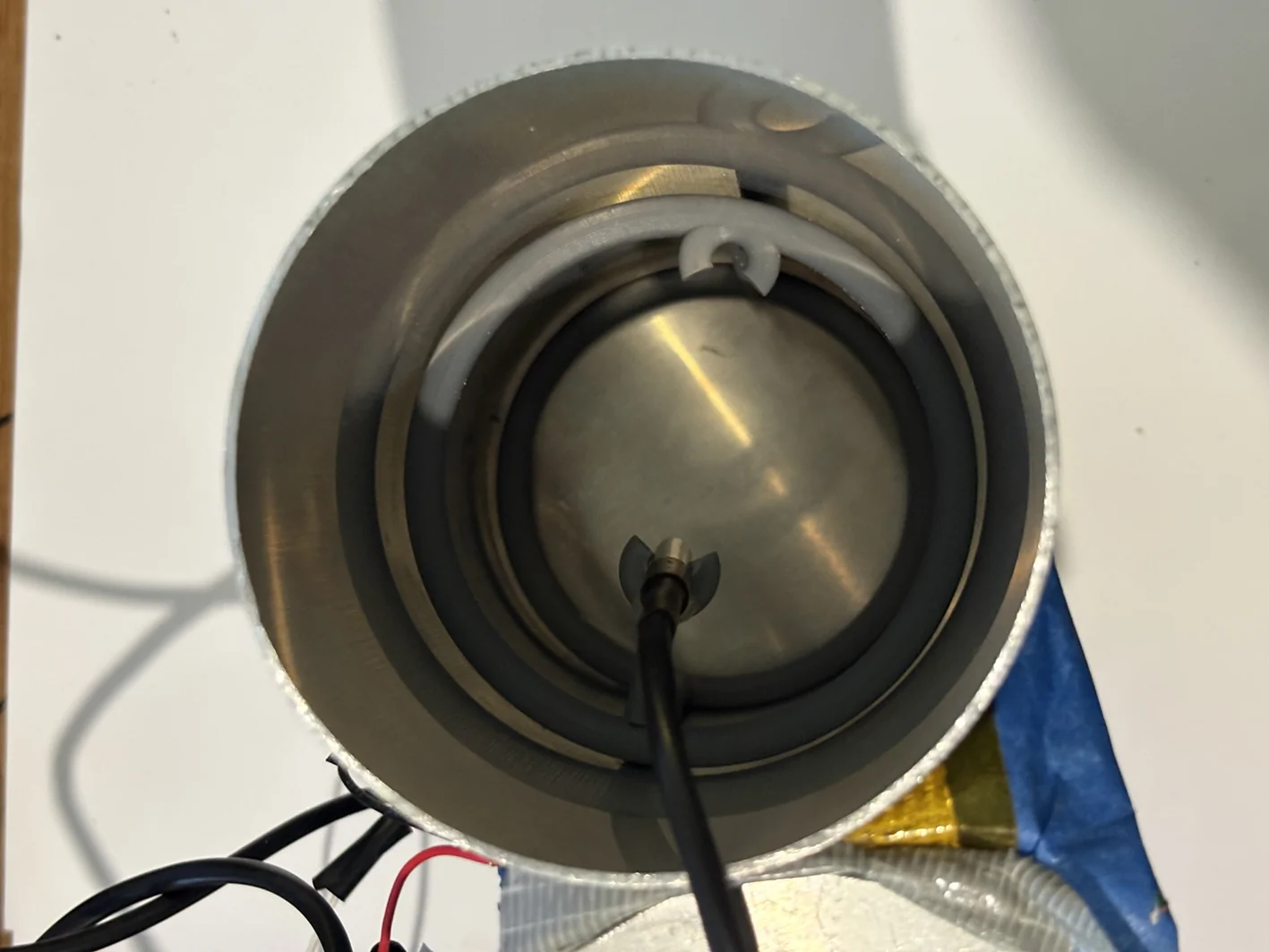
An internal view of the aluminum cylinder with a temperature sensor hold showing heating cloth on side.

Fig. 21Side, bottom, and overhead views of the cylinder with the heating cloth. The heating cloth must be in contact with the top and bottom of the thermos.Fig. 21

**(a):**
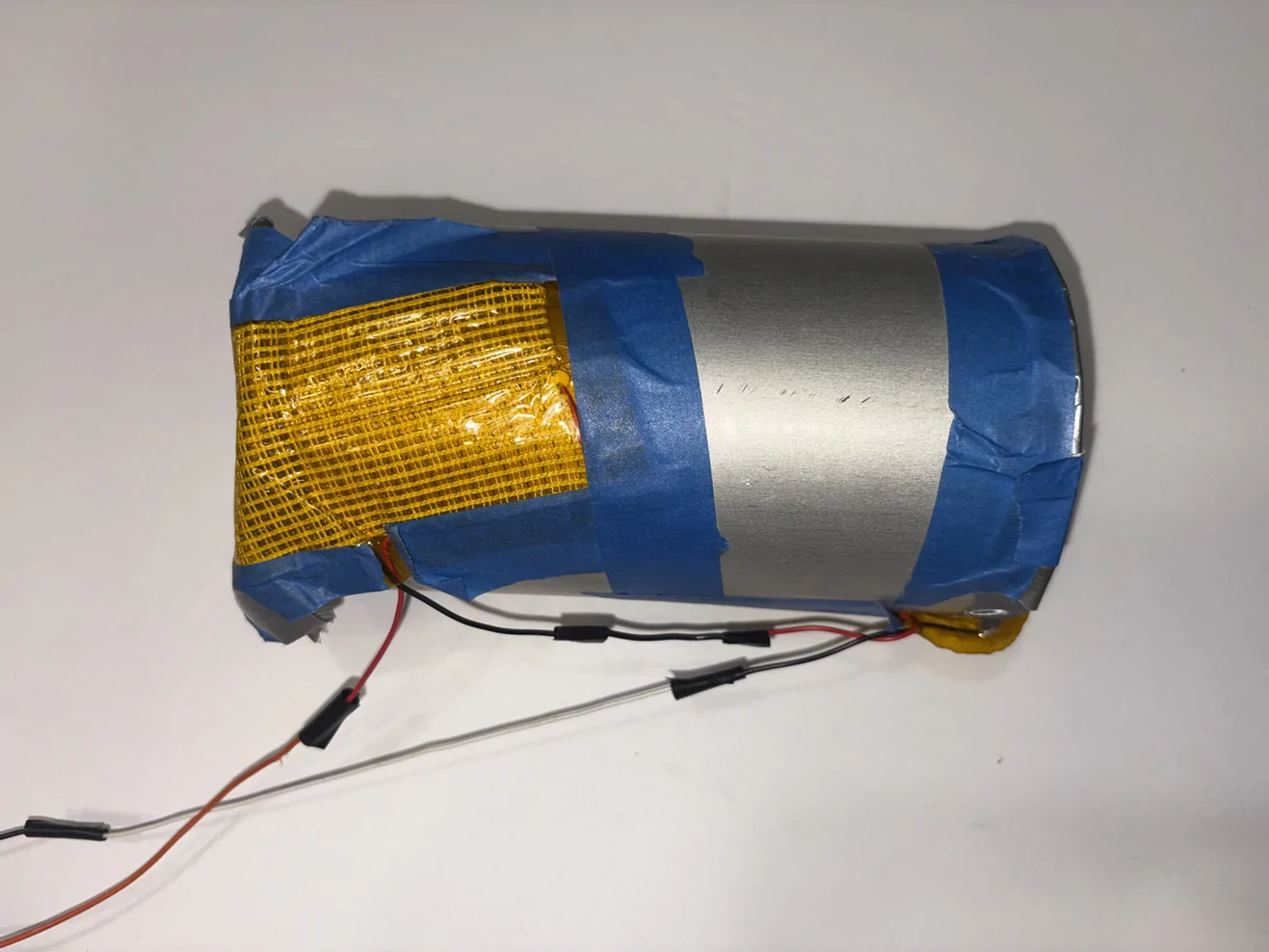
.

**(b):**
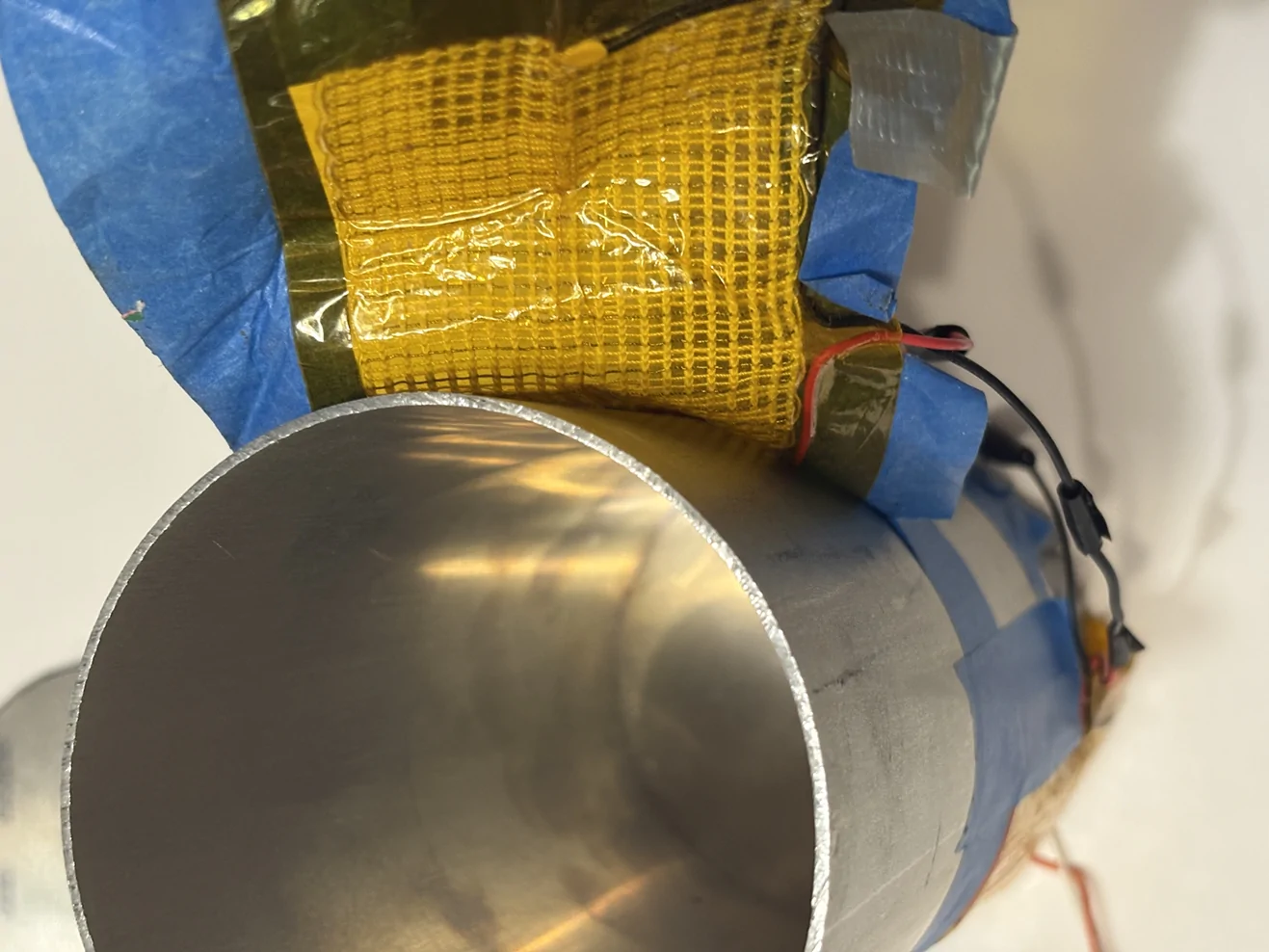
.

**(c):**
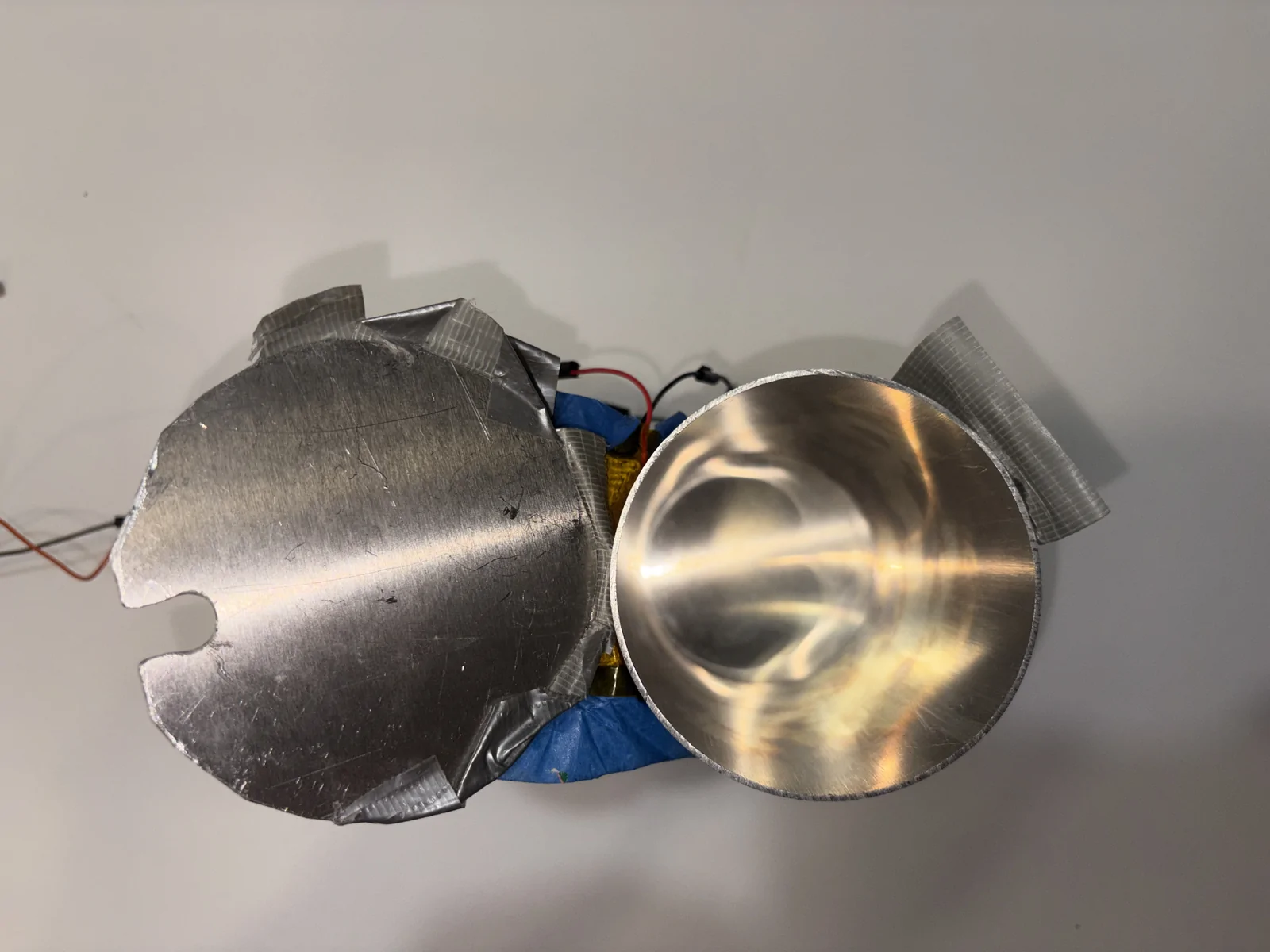
.

### 4.7 Assembly notes and tips

• Ensure that the digital thermometer is not in direct contact with the heater board. It may be preferable to position the heating cloth along the curvature of the thermos while gluing the thermometer to the lid.
• You may wish to make a culture holder for whatever your culture material is.

### 4.8 Electrical tests

Electrical tests are divided into two parts: 1. the heater board and 2. the controller.

### 4.9 Electrical measurements

Measure the following electrical parameters by serial number. Investigate and correct any abnormal measurements before applying power. Document any components that require replacement.

Begin with no connections to the DUT (Device Under Test).

• **Power Jack:** Measure resistance to the ground at J101 center pin. The measurement should indicate an open circuit or a resistance greater than 1 mega ohm.
• **Heating cloth:** The resistance should measure approximately 4.2 Ω.

### 4.10 Design decisions

We hope to enable the incubation of 3M Petrifilm samples in remote locations to support the quantitative measurement of E. coli in potential drinking water for Engineers Without Borders. However, researchers may use the Moonrat II to portably incubate almost any biological processes that require controlled warming from a 12 V power source. For example, a larger incubation chamber could be used to incubate the eggs of large birds or reptiles.

In fact, our choice of using a microcontroller instead of a simple thermostat potentially supports preprogramming incubation regimes which are more complex than a steady target temperature. Our largely unused STRATEGY_FUZZY is an example of an approach where a complex control strategy could be built, but not easily implemented with a thermostat. The system was intentionally designed to be modular in order to accommodate such alternative applications.

Although Bluetooth and Wi-Fi are commonly used for device control, these technologies would introduce additional complexity and potential points of failure. They are inconsistent with the design goal of producing a rugged, portable tool suitable for deployment in communities without reliable internet access.

We have chosen the Big Boss Thermos because:

• it is large enough to incubate Petrifilms,
• it is sturdy and provides excellent insulation, and
• its cap could be drilled with minimal damage and has room for mounting the heaterboard PCB.

### 4.11 Use of an internal cylinder

The decision to use an internal aluminum cylinder to distribute heat was driven by the observation that the internal temperature depends on proximity to the heating cloth. When the heating cloths are wrapped around an aluminum container, the temperature becomes evenly distributed throughout the cylinder (see the Validation section below).

A conventional solution would be to use a small fan which circulates air within the chamber. This would add cost and complexity, however, and would increase the risk of failure. Even worse, the air flow in the chamber would depend on what is placed inside the device, whereas this solution ensures that the more thermal mass is placed in the chamber, the more consistent the heating will be. Not having any moving parts is a huge advantage for reliability.

## 5 Operation instructions

### 5.1 Acquiring and powering

The Moonrat II should be powered by a 12 V plug in the standard 5.5 mm × 2.1 mm size. While a wall-mounted power adapter may be used, the primary advantage of the Moonrat II is its portability. It is typically powered by a 12 V lithium-ion power bank such as those manufactured by TalentCell™. These claim to be 6000 mAh at 12 V, or 72 watt-hours. Since some operating conditions allow the Moonrat II to consume less than 5 W, a single battery pack can provide up to approximately 14 h of continuous use. By using multiple power banks and optionally recharging them with a solar charger, a user can achieve 48 h of powered incubation time. This is the longest incubation time for some applications.

Because the Moonrat II relies on widely available external batteries, users must plan their battery usage in advance. To assist with battery management, the Moonrat II displays the amp-hour consumption since it was last turned on.

### 5.2 Three button menu interface

The Moonrat II features an OLED display and three control buttons, as shown in Fig. 12. The button functions are “UP”, “DOWN”, and “SELECT”. The “UP” and “DOWN” buttons allow users to navigate the short menu, set temperatures (Fig. 23), and manage duration (Fig. 24). The “SELECT” button allows users to make menu selections and back out of sub menu options as shown in Fig. 22.

There is a short “beep” each time a button press enacts a legitimate command, informing the user that it was effective.

**Fig. 22:**
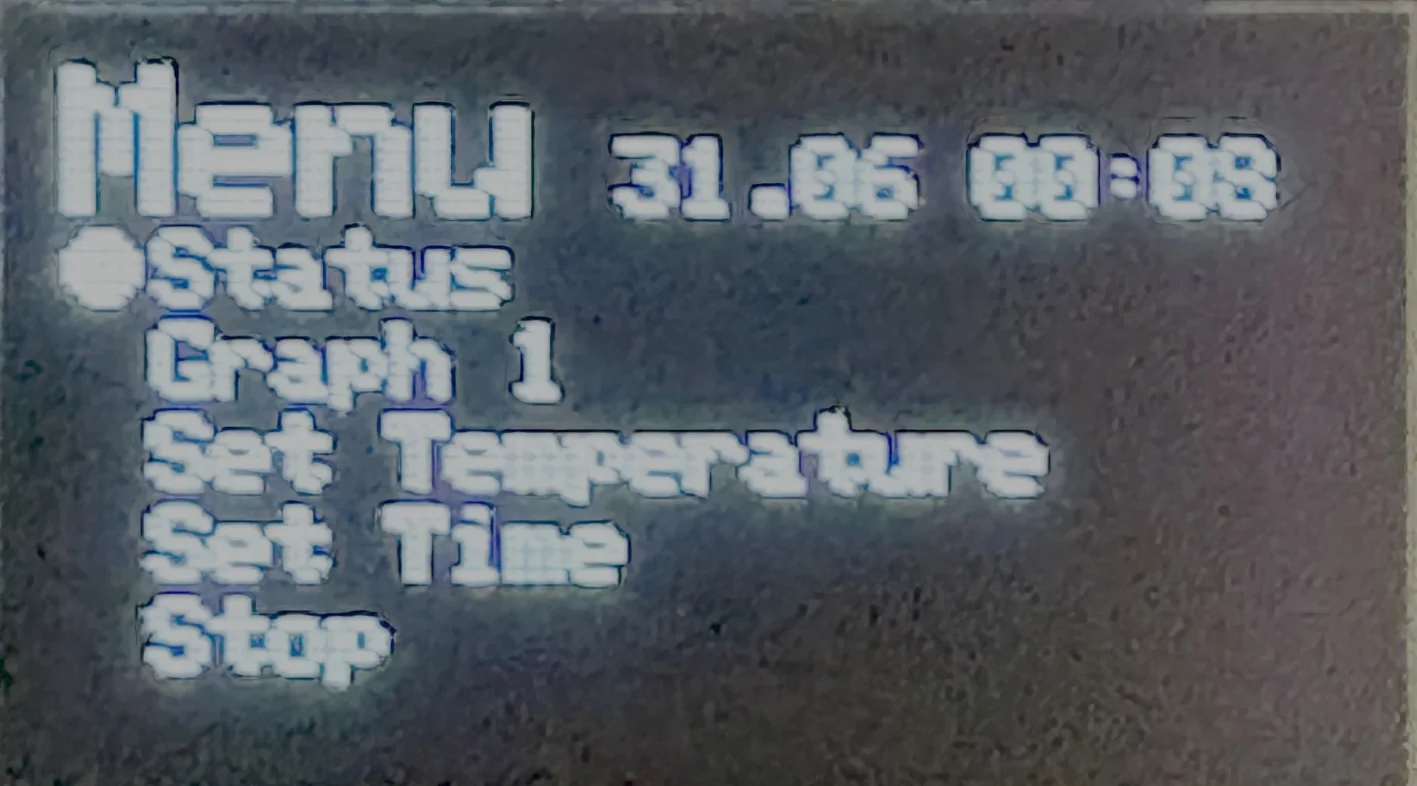
Photo of the OLED showing the main menu.

**Fig. 23:**
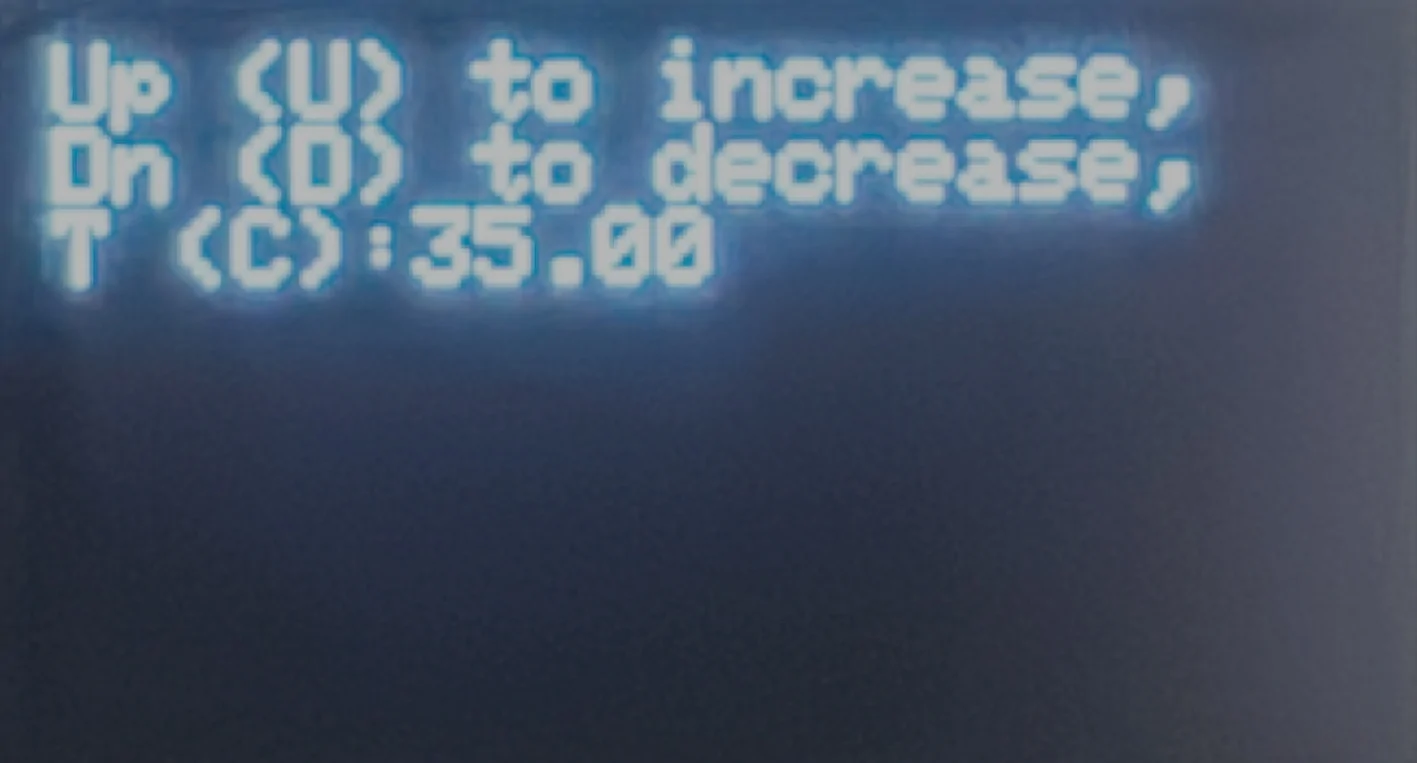
Photo of the OLED showing set temperature menu.

### 5.3 Choosing incubation temperature

The “Up” and “Down” buttons on the OLED display can change the target incubation temperature by 0.5 C per press. The digital thermometer in the Moonrat II has an accuracy of 0.5 C.

### 5.4 Starting and stopping

The incubation proper begins when a user enters the “start” command in the menu. During incubation, this command becomes “stop”. The menu option is thus a toggle for the operation.

### 5.5 Incubation time

**Fig. 24:**
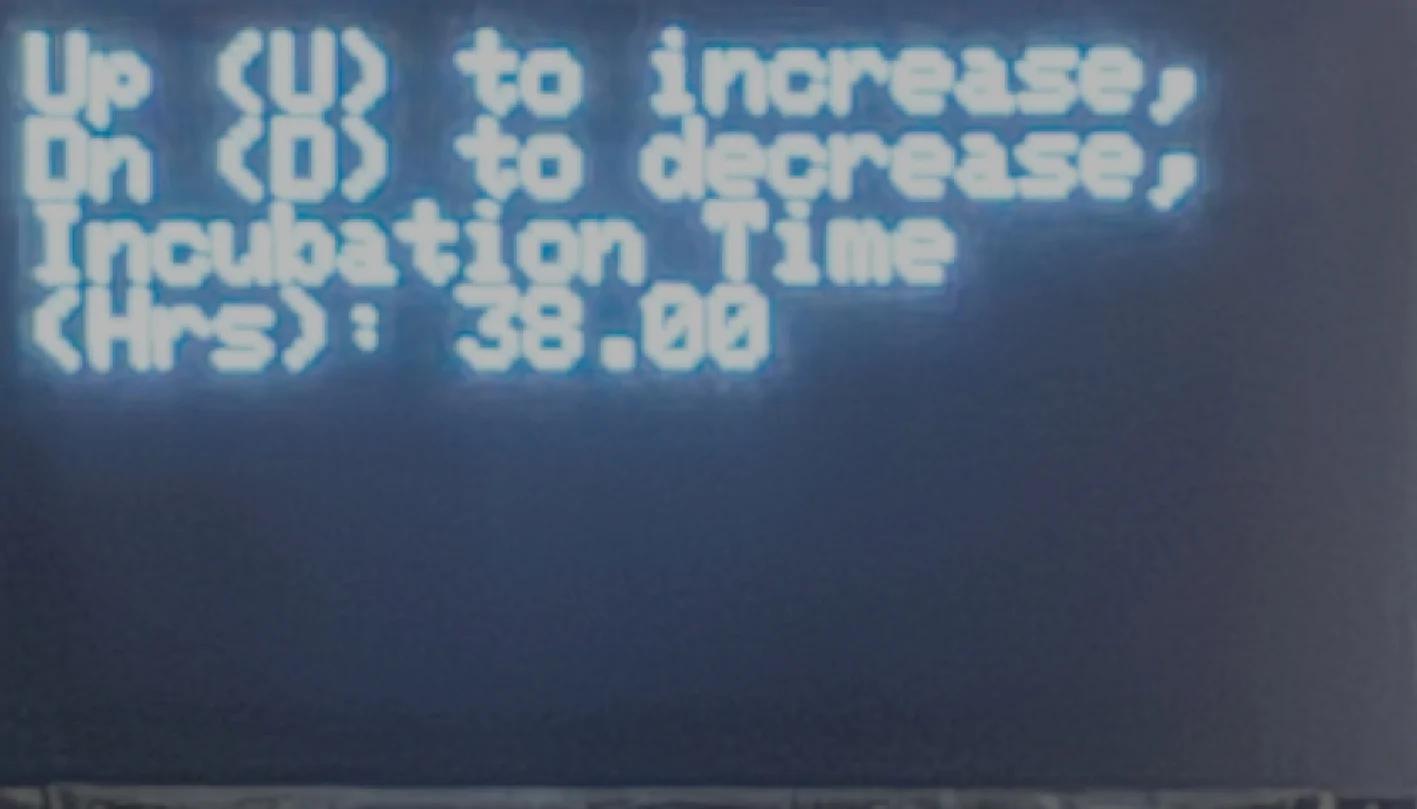
Photo of OLED showing incubation time control screen.

### 5.6 Review graph

The OLED screen displays a graph of past temperatures. This graph allow the user to verify that the correct temperature has been maintained (and entered) as depicted in Fig. 25.

**Fig. 25:**
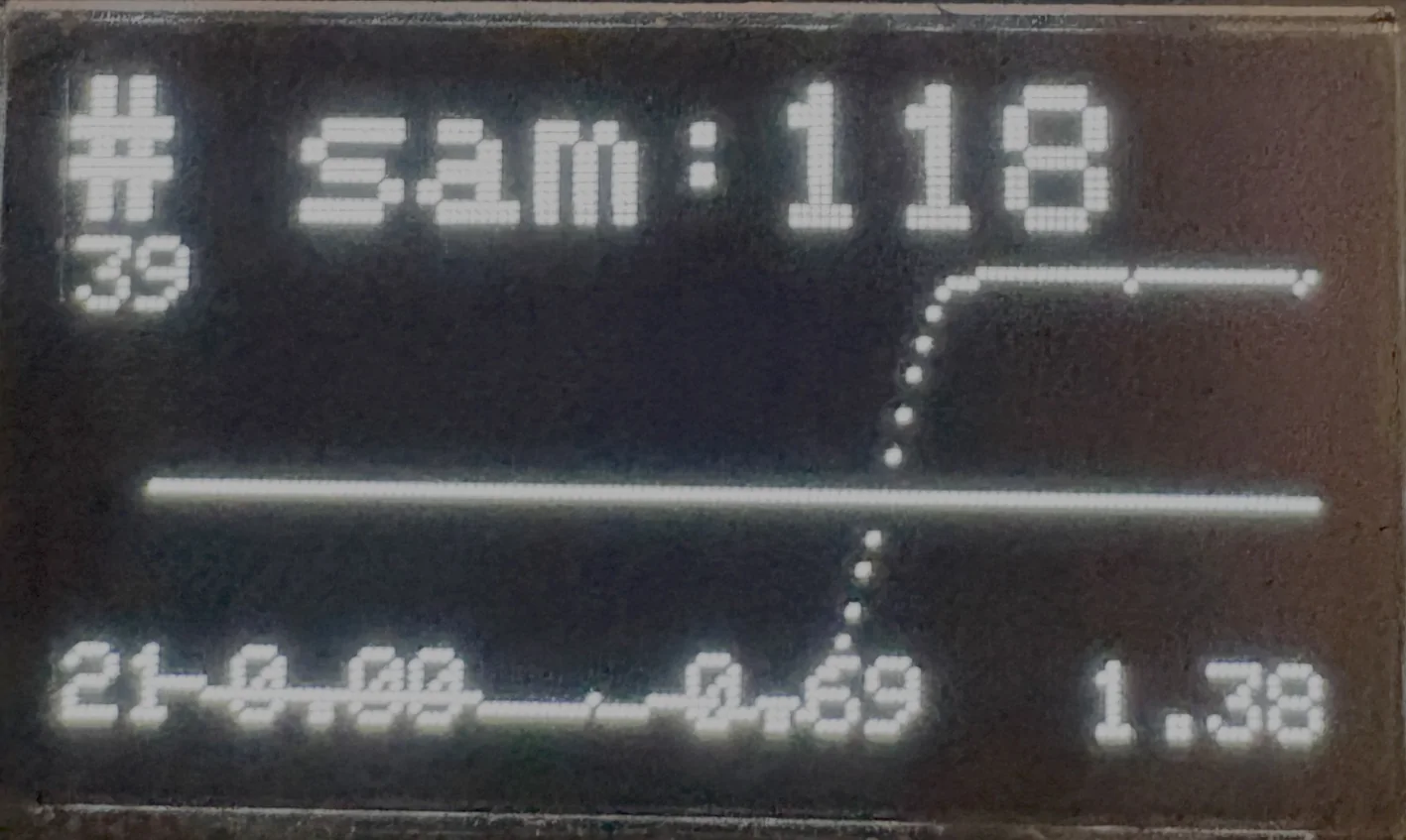
Photo of review graph showing Warmup.

### 5.7 Status

The status screen, as depicted in Fig. 26 shows:

• The current temperature
• The energy (in watt hours) used in the current experiment
• The amp hours used in the current experiment
• The time in hours of the experiment

The “Up” and “Down” buttons can be used on this screen to modify the Calibration Temperature by tenths of a degree (°C) (see Calibration Procedure in Section 5.16).

**Fig. 26:**
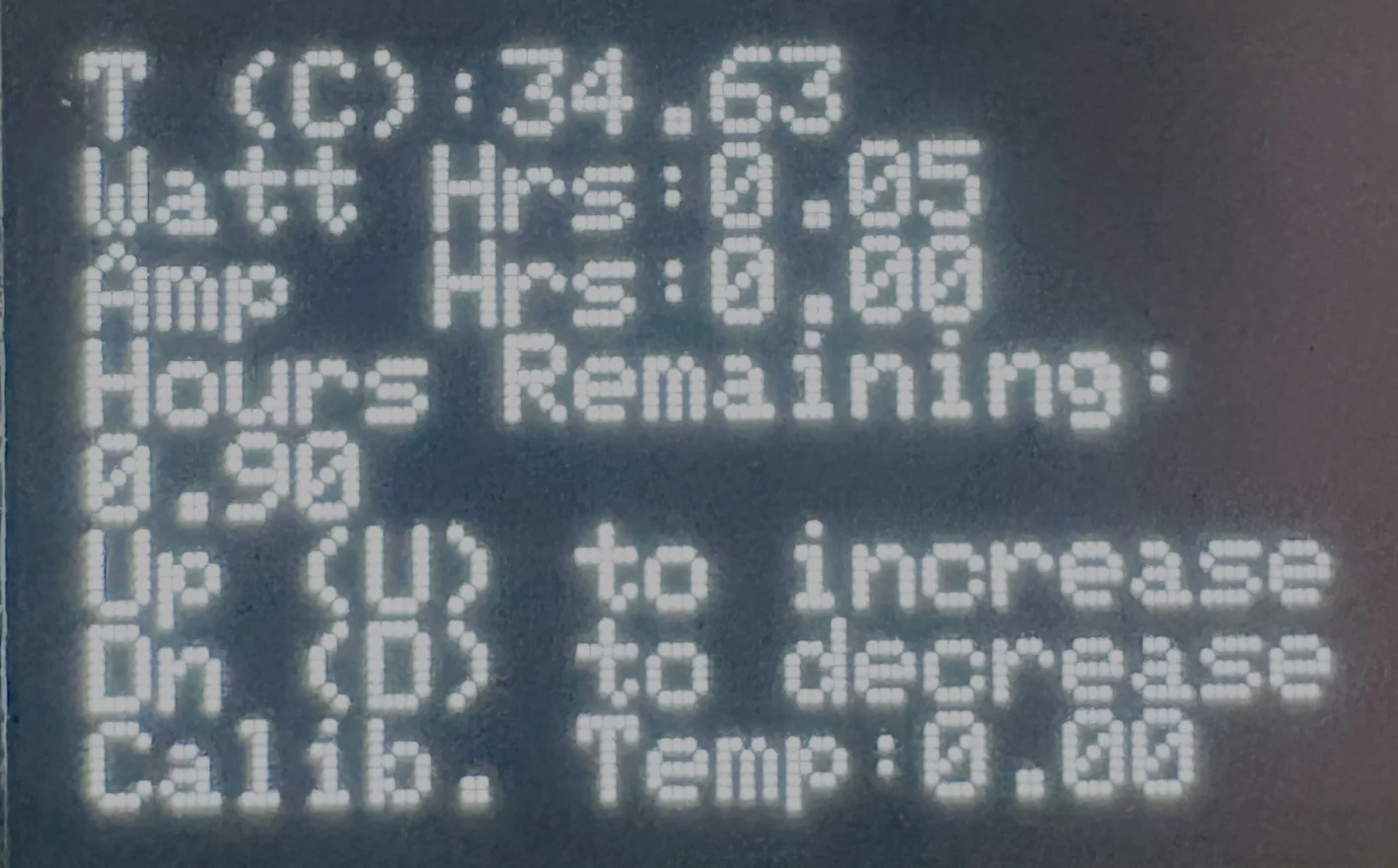
Photo of example status screen, showing calibration options.

### 5.8 Planning battery life

The “Status” screen shows the current watt hours and amp hours the Moonrat II has used since last powered on. The temperature history per minute is stored in the EEPROM and persists across power resets, such as changing the battery, but the watt hours statistics do not.

The Moonrat II also reports the amp hours that the current experiment has used. With this information, users can predict the Moonrat II’s remaining battery life so long as they recorded the battery charged capacity at the beginning of the experiment. Although one can plug the Moonrat II into any 12 V power source, the expected power sources are portable “power bank” rechargeable batteries. They typically have a stated lifetime of 6000 milliamp-hours, though this number should be viewed skeptically. Nonetheless, given such a fully charged battery, if the current ambient temperature vs. incubation and insulating power of the temperature chamber show a utilization of 4 amp hours and 8 h of wall-clock time, it is likely that the battery will run out before the next 4 h of wall-clock time. To achieve an uninterrupted incubation, a convenient solution is to have multiple, fully-charged power banks on hand for the Moonrat II’s use. This will, however, make the use of the Moonrat II somewhat more expensive and less portable. One current brand of 6000 milliamp hour battery pack weighs 384 g. Please see Section 6.6 for more information.

### 5.9 Changing the incubation chamber

The authors discovered the need for the Moonrat II while working as volunteer engineers for Engineers Without Borders (EWB). One way that EWB assays water quality is with 3M Petrifilms. The “Big Boss” Food Jar by Thermos, described in this paper, is a good size for incubating up to 40 Petrifilms at a time with a proper internal tray. However, there is no reason one could not use a larger or smaller incubation chamber. The “heater board” module, with the heating cloth and digital thermometer, can be placed in a much larger chamber to incubate different media. For example, incubating a test tube rack or 125 mm petri dishes would require a large chamber. This would not affect the operation of the Moonrat II, other than it may require more power to warm a larger chamber. A very large chamber might reach incubation temperature with the Moorat’s maximum, full power of 24 W.

### 5.10 Dumping recorded data via the serial port

The Moonrat II can connect to a serial port monitor, such as that provided by the Arduino IDE, at 115200 baud. Transmitting an “x” on this serial port will cause the Moonrat II to report its full memory of recorded temperature samples. Reset the ROM by sending an “r” for “reset” to the Moonrat II.

### 5.11 Choosing the temperature control strategy

We coded and tested the Moonrat II software with the Big Boss vacuum flask and three distinct strategies, which a user can select in the code in file utility.h on lines 40–42. This of course requires a recompilation and re-upload.

The STRATEGY_THERMOSTAT is a simple thermostat. The STRATEGY_PID uses a standard Arduino proportional–integral–derivative controller. The STRATEGY_FUZZY strategy offers flexibility and control if a programmer is willing to configure it for their own purposes.

Each of these strategies has been tested and, as far as we can tell, they all work fairly well. However, we prefer and have validated STRATEGY_PID most extensively as requested by the reviewers of this paper. Multiple strategies have zero value to a user who is not taking advantage of the open-source nature of the Moonrat II. However, one of the advantages of open-source systems is the flexibility to customize the system for unpredictable future needs. The fuzzy controller and the simple thermostat strategies are included and documented for that reason.

### 5.12 Fuzzy controller strategy

The STRATEGY_FUZZY strategy is fully documented in https://zenodo.org/records/20531233/preview/moonrat-1.0.4.zip?include_deleted=0#tree_item495.

### 5.13 Thermostat controller strategy

The thermostat controller strategy is very simple: if the temperature is too low, turn the heater on; if it is too high, turn it off.

Simplicity is an advantage. However, this strategy tends to oscillate unnecessarily around the correct strategy. The thermostat may slightly overshoot the temperature due to the delay in equalizing the temperature around the sensor with the mean temperature in the chamber, for example. However, the thermal mass of the sample will tend to cause its temperature to vary less than the reported temperature of our sensor, mitigating oscillation.

### 5.14 PID controller strategy

A Proportional–Integral–Derivative (PID) controller solves the “overshoot” problem. This is the classic way to control temperature, and in our tests, it has always been adequate. We have tested more with the PID controller than any other, and recommend it. The relatively slow heat transfer time through the internal aluminum cylinder requires us to use an “anti-windup” tuning. The Fuzzy Logic Controller has no real advantage over this controller when it comes to normal incubation, but may have some advantage in future incubation regimes (where temperatures change over the course of the incubation).

### 5.15 Completion of the incubation

The Moonrat II begins beeping loudly and stops heating when the end of the incubation period is reached.

### 5.16 Calibration procedure

The DS18B20 temperature sensor is waterproof, making it relatively easy to calibrate. The manufacturer claims it does not need to be calibrated, and we have never observed one out of calibration. Nonetheless, it can be calibrated in an ice bath. A description of how to prepare the ice bath can be found online. The serial port output of the Moonrat II with the Arduino IDE will print the temperature every 30 s. If the temperature does not stabilize to 0.0°C, you may enter a calibration temperature adjustment in tenths of a degree °C on the Status Screen using the up and down buttons. If the temperature sensors read ice water as less than 0.0 by x tenths of a degree, then enter a positive value of x as the calibration temperature.

This thermometer is not rated for boiling temperatures, so it is not practical to do a more sophisticated two-point calibration.

## 6 Validation and characterization

### 6.1 User validation during operation

The OLED displays a graph of temperature. This allows the user to dynamically observe the temperature inside the chamber over the last 2 h to assure that the operation has maintained the correct, uniform temperature. Additionally, if you attach a terminal to the USB-C port of the minima and type “x”, it will dump all of the stored temperature data as one sample per minute during the current incubation, no matter how long.

### 6.2 Temperature uniformity

The fundamental operational performance parameters of an incubator is the ability to keep the incubated sample at the correct temperature for the correct amount of time. Ideally the interior of the incubator would be exactly the same temperature at all times, so that any sample placed anywhere in the chamber would be at the correct temperature. In order to measure this, we built a “cage” (Fig. 27) out of hardware cloth and ordered two highly precise Innovation Sensor Technology (TiSC 716) temperature sensors. These were calibrated with a warm water bath at 35°C to the value given by the control temperature sensor of the Moonrat. The sensors were small enough that they could be placed in various positions within the already small chamber. To accomplish this mounting, we made a “cage” out of hardware cloth that allowed us to zip tie the sensors into different radial and axial positions. The results are shown in Table 4.

One might think that “stack effect” would cause the higher part of the chamber to be warmer. In fact, the impact of the stack effect in such a small chamber made of metal is very small. We have demonstrated this fact by turning our dual-sensor instrumentation upside down, and noting there is almost no change even after 30 min.

**Fig. 27:**
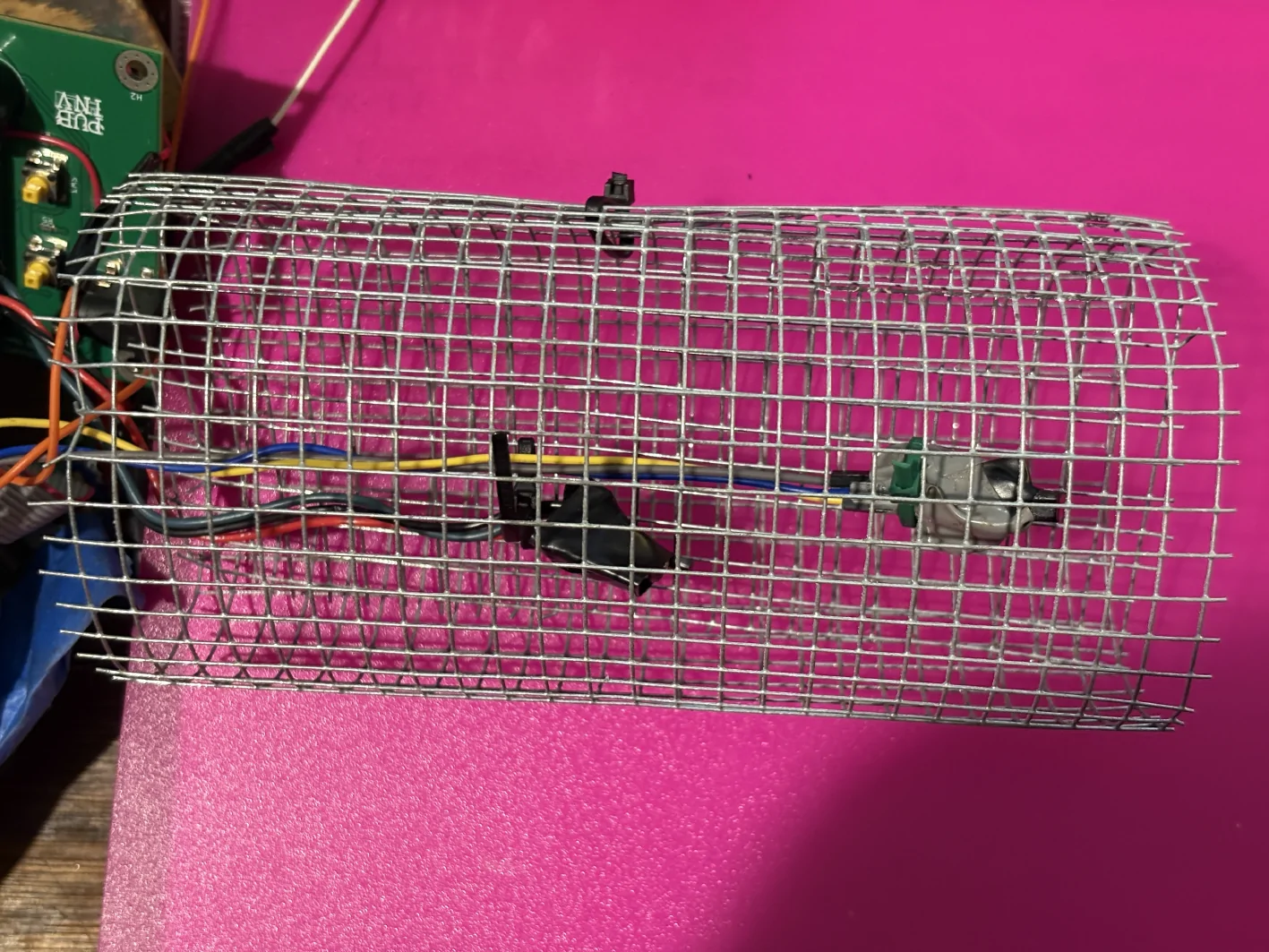
Temperature test cage.

However, the proximity to the heat source DOES effect the temperature, which led us to adopt the rather complicated “wrap-around” approach with our heating cloths. Before the current design, which places the heating cloths in contact with the axial ends of the cylinder, there was as much as a 1.25°C difference within the chamber. The table below gives the data for various sensor positions (all based on the latest “wrap-around” design).

Trails G and H were made inside the Thermos; the other trials were made with the aluminum cylinder outside the Thermos, powered directly with a desktop power supply.

Trial H demonstrated the extreme radial difference, with one sensor in the center and one near the edge, both in the middle of the cylinder axially.

The greatest observed temperature difference was 0.64°C. This compares favorably to the stated Temperature Uniformity of some commercial incubators, such as the Benchmark Scientific myTemp™ Mini, heat only [13]. This is a “worst case” analysis, since the temperature sensors are very small, were hanging in the air, and were only in contact with electrical tape. In actual practice, a biological sample would have a much greater thermal mass and would probably have a relatively high moisture content, which would tend to make them heat conductive. Thus they would tend to even out any spatial temperature differences they would encounter. Since most biological samples, such as E. coli Petrifilms, have a specified tolerance of ±1°C, the Moonrat II should be accurate for most applications.

**Table 4:** Temperature uniformity test.

Experiment	Center Temp. (°C) #1	Extremal Temp. (°C) #2	Difference (°C)
A: Extreme at high (close to wire entrance) position, 10 V	35.75	35.45	0.30
B: Extreme close to wire entrance, but turned upside down, 10 V	35.95	35.65	0.30
C: Extreme far from wire entrance, lower part resting on ${\frac{1}{4}}^{\prime \prime }$ foam core, 10 V	35.68	35.12	0.56
D: Extreme far from wire entrance, resting on plywood, 10 V	35.33	34.67	0.64
E: Extreme far from wire entrance, resting inside thermos, 2.5 V, 0.19 amps	35.13	34.72	0.41
F: Same as E, but turned upside down, 3.1 V, 0.21 amps	33.84	33.82	0.02
G: Extreme at wire entrance (high), inside thermos, 3.2 V, 0.25 amps	34.38	33.81	0.57
H: Extreme at the same level as control (middle) but as close to the radial edge as possible	35.16	34.96	0.20

### 6.3 Power utilization

Not counting the heat output, the power draw of the OLED is approximately 20 mA (though it depends on what is displayed). The Arduino R4 Minima nominal maximum current draw is 37 milliamps [16]. The total power consumption of the control system is thus expected to be approximately 60 mA at 12 V, or about 0.72 W, although we have observed it to be closer to 0.25 W.

The software outputs the average wattage used by the system to maintain incubation temperatures. This is directly related to the insulating power of the incubation chamber, and the difference between the ambient and the incubation temperature.

At present, incubating at 35°C against an ambient temperature of 25°C using the “Big Boss” Thermos is measured as 1.1 W. Adding the nominal 0.72 W to this, the expected power draw in this scenario is 1.82 W. The expected battery life can be computed as shown in Eq. (1):

(1) $$L=\frac{C\times V}{1000\times P}$$

where:

• L is the expected lifetime in hours
• P is the power consumption in watts
• V is the battery voltage (almost always 12 in practice)
• C is the battery capacity in milliamp-hours

Note that battery capacities are notoriously exaggerated, and should be looked upon skeptically in predicting battery lifetimes. Nonetheless, this formula has been found to be generally useful. In our Bill of Materials we have suggested a brand, but we do not consider that a part of the Moonrat II itself—you can use any 12 V battery. You can choose the size to match your incubation time and expected power usage (based on ambient temperature and incubation temperature), keeping in mind that larger batteries are a bit harder to carry in a portable application.

### 6.4 Battery life and temperature control at room temperature

Using the PID controller strategy in normal room temperature air of approximately 25°C, the TalentCell battery (nominally 6 amp-hours) lasted 50 h and 44 min, as depicted in Fig. 28. This is slightly longer than some 48 h incubation regimes, such as that of the E. coli Petrifilms. More specifically, this battery claims a 66.66 watt hour capacity. Converting to watt minutes and dividing by 3044, we obtain an average measured 1.31 W of power consumption to maintain 35°C against 25°C ambient air (and to maintain the power to the microcontroller).

The temperature overshoot shown at the beginning of Fig. 28 has been repaired by implementing an anti-windup change in the integrative tuning, which is depicted in Fig. 29

In the 48 h run measured via the log reported for the evaluation against the desktop incubator in Section 6.7, the following temperature statistics were observed:

**Fig. 28:**
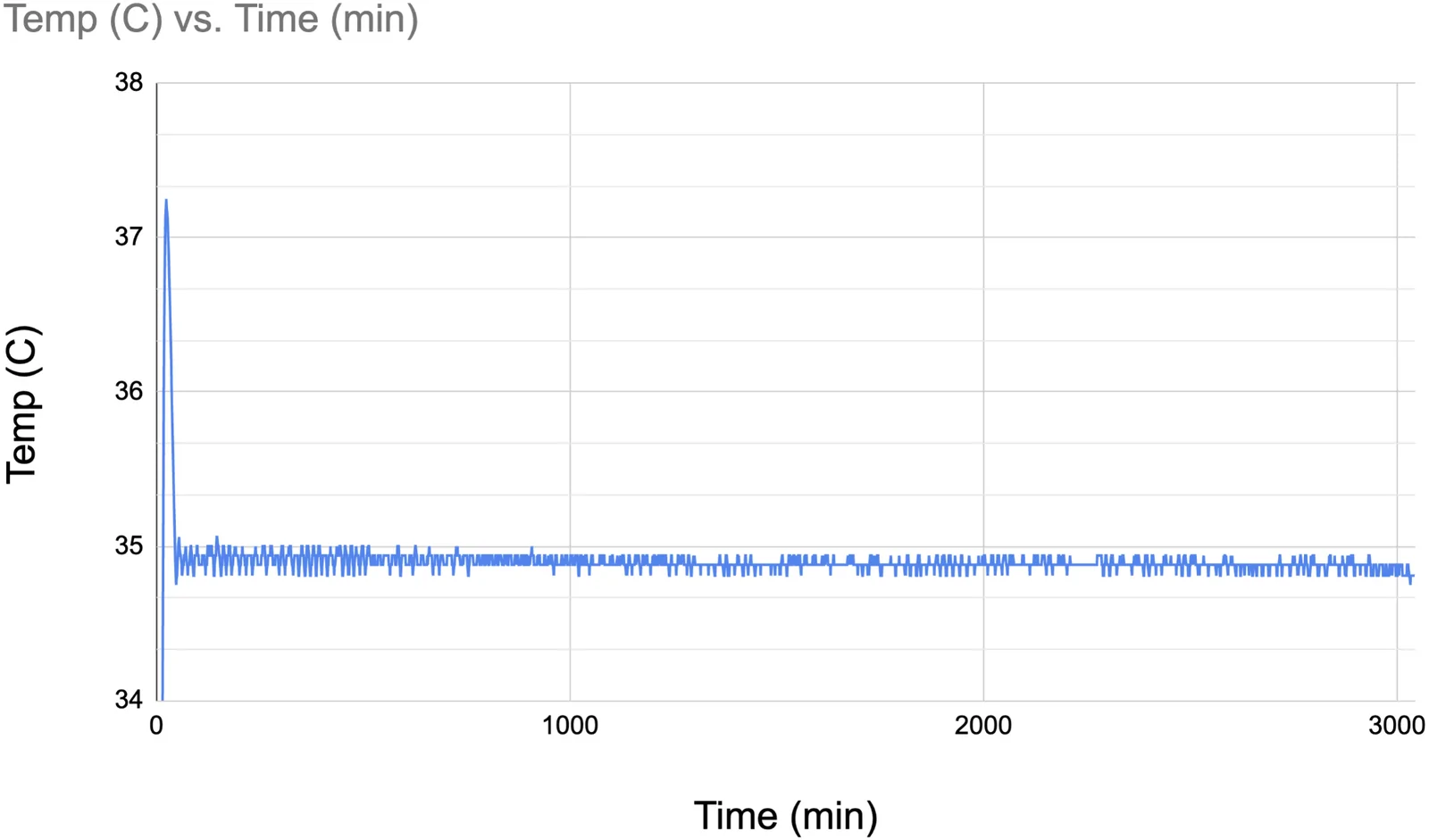
50 h, 44 m battery life at 35 C at ambient room temperature.

**Fig. 29:**
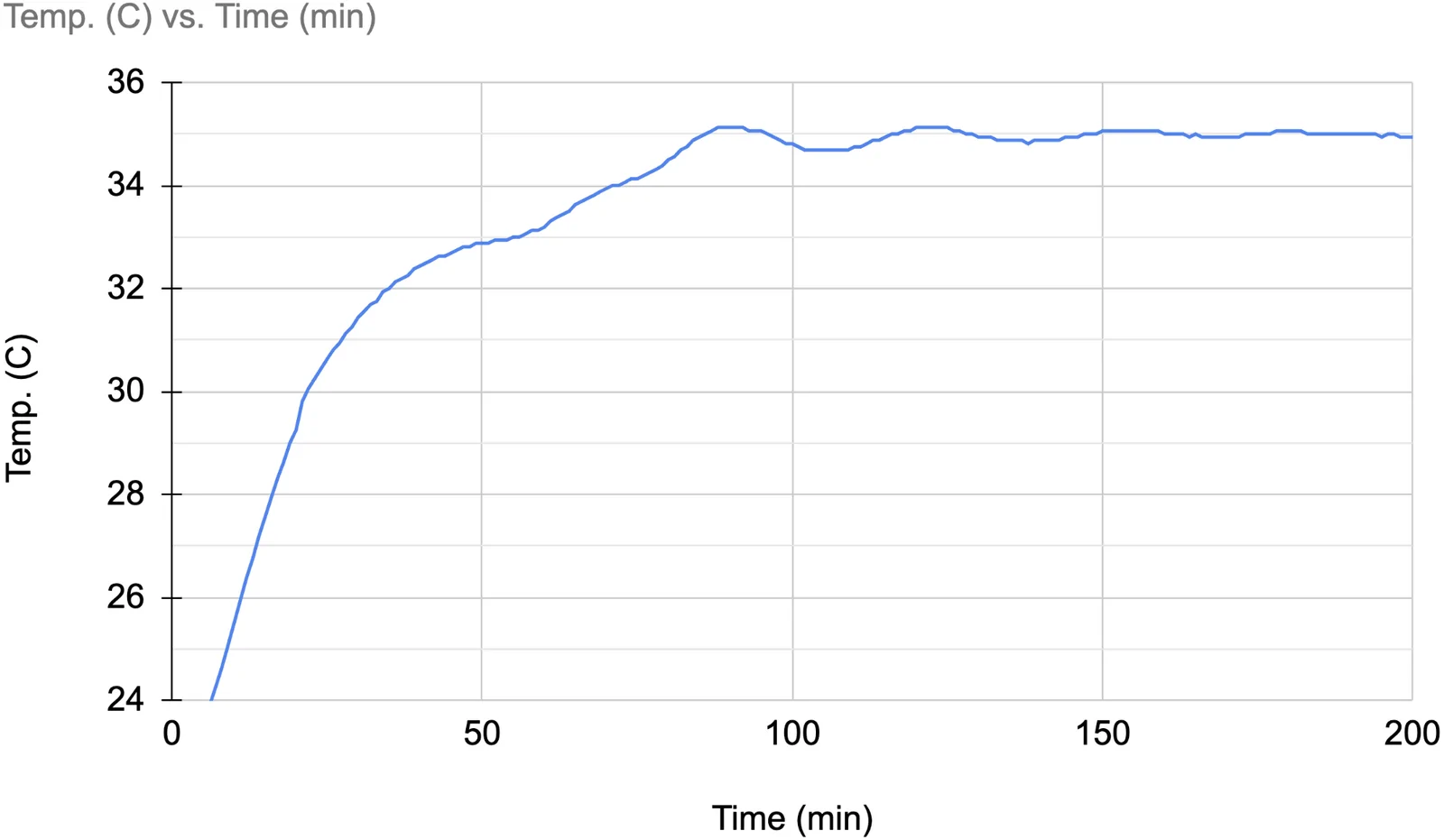
Temp. vs. time for 3.5 h with proper PID tuning.

• Settling time: 94 min
• Steady-state range after settling: 34.50–35.25°C
• Steady-state mean: 34.997°C
• Steady-state standard deviation: 0.040°C

### 6.5 Battery life in a refrigerator

As an extreme test of the Moonrat II, it (and its battery pack) were placed in a refrigerator at 4.2°C while incubating at 35°C. This is much colder than human beings can generally tolerate—even inside a tent in the mountains humans would tend to create a warmer environment. In the refrigerator, the Moonrat II ran for 19 h on a single battery charge. This represents a total power usage of 3.5 W.

### 6.6 A linear model of power consumption

Treating heat loss as proportional to temperature difference and fitting the two experiments reported above, a model of power consumption to maintain incubation is approximately:

(2) P(D)≈0.25+0.1D

where P is in watts and D is in °C in Eq. (2). This approximate formula could be used to predict battery life for a given incubation condition where D is the expected difference between the ambient air and the incubation temperature. If the incubation regime is H hours, the needed battery capacity of a 12 V battery in amp-hours would be:

(3) $$A=\frac{P\left(D\right)\cdot H}{12}$$

where A is in amp-hours in Eq. (3). This may be useful for sizing batteries, but actually practicing with an empty incubator before committing biological samples would be advised if possible.

### 6.7 Comparison with a desktop incubator

In order to compare the Moonrat to a basic competitor, we purchased a Thermo-Scientific Heratherm™ 18L benchtop laboratory incubator and designed an experiment. We determined that less than a 10% difference in E. coli colony forming units (CFU) counts could be considered functionally equivalent between the two systems.

We collected a jar of water from Waller Creek, which runs through downtown Austin and had previously demonstrated E. coli CFU counts of about 7 CFU per milliliter. We inoculated 14 Petrifilms from the same jar and placed 7 in the Moonrat II and the Heratherm each.

Unfortunately, the creek water was cleaner than when we last measured it. The results between the incubators were consistent, but there was insufficient statistical power to draw any conclusions. Nonetheless, the results are depicted in Fig. 30.

To obtain more reliable results, we ordered an agar slant of an E. coli culture from Carolina Biological Supply. After testing for an approximate CFU Count per dilution, we inoculated a 1 L saline solution with a scraping from the slant and made a common suspension. Fifteen films numbered $A_{n}$ and $B_{n}$ were inoculated pairwise, and we stirred the suspension before each 1 ml pipette draw. Fifteen films were then incubated in the Heratherm (A) and the Moonrat II (B). Mean colony counts were 58.8 CFU (SD 17.2) and 54.7 CFU (SD 20.1) for incubators Heratherm (A) and Moonrat II (B) respectively, corresponding to an observed mean ratio of 1.076. We detected no statistically significant difference between incubators (Standard t-test: t = 0.61, p = 0.55). Plate-to-plate variability was substantial, however, and a 90% confidence interval for the ratio of mean counts was approximately (0.884,1.321); consequently, the experiment did not establish equivalence within either a ±10%. This high variability may reflect heterogeneity of the inoculum prepared from an agar-slant culture.

**Fig. 30:**
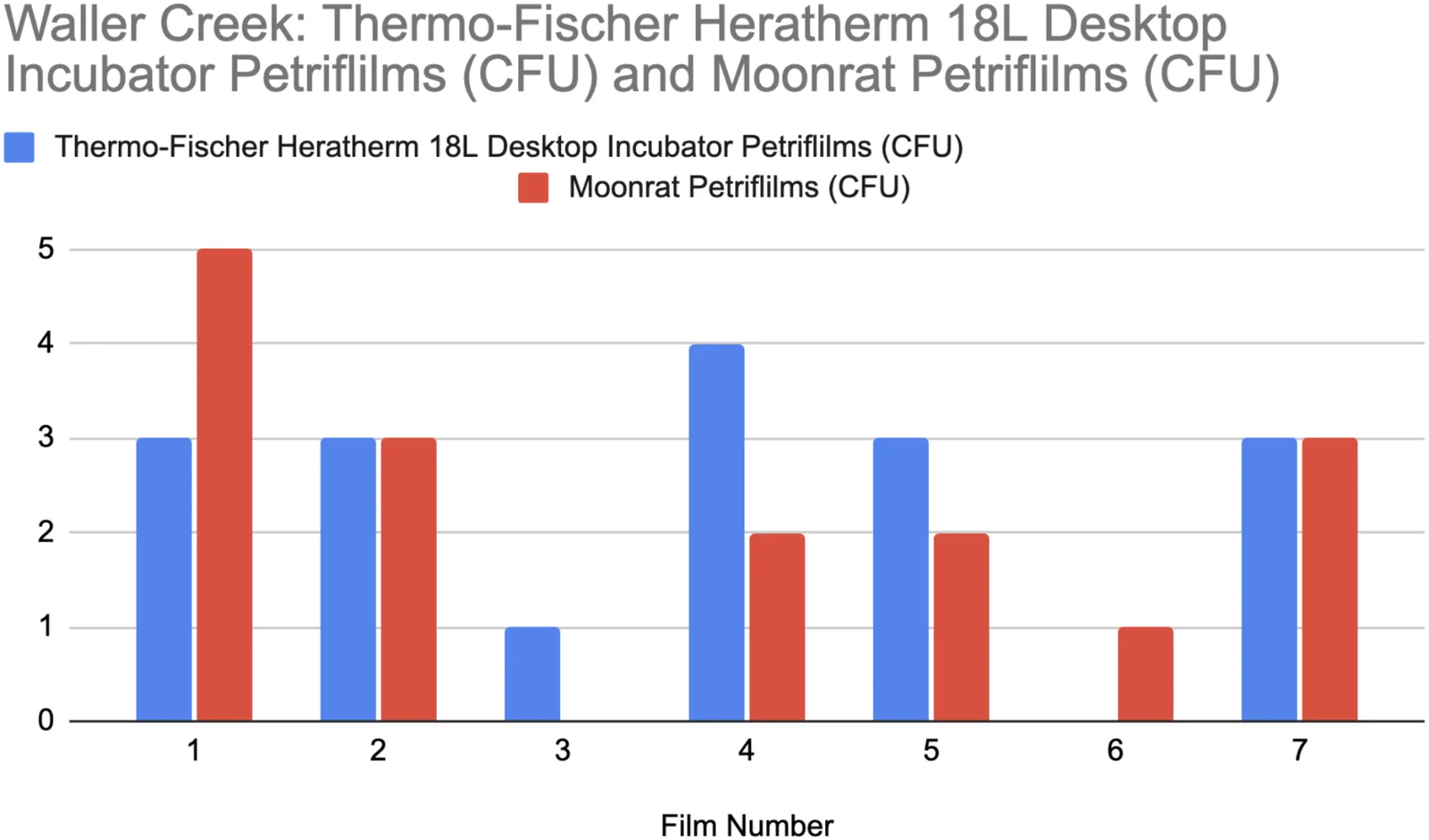
Waller creek comparison.

In summary, although the difference between the means of the two incubators was less than 10% (mean ratio 1.076), we could not statistically prove either that the incubators were equivalent or different. A bar chart representing the CFU counts is shown in Fig. 31.

**Fig. 31:**
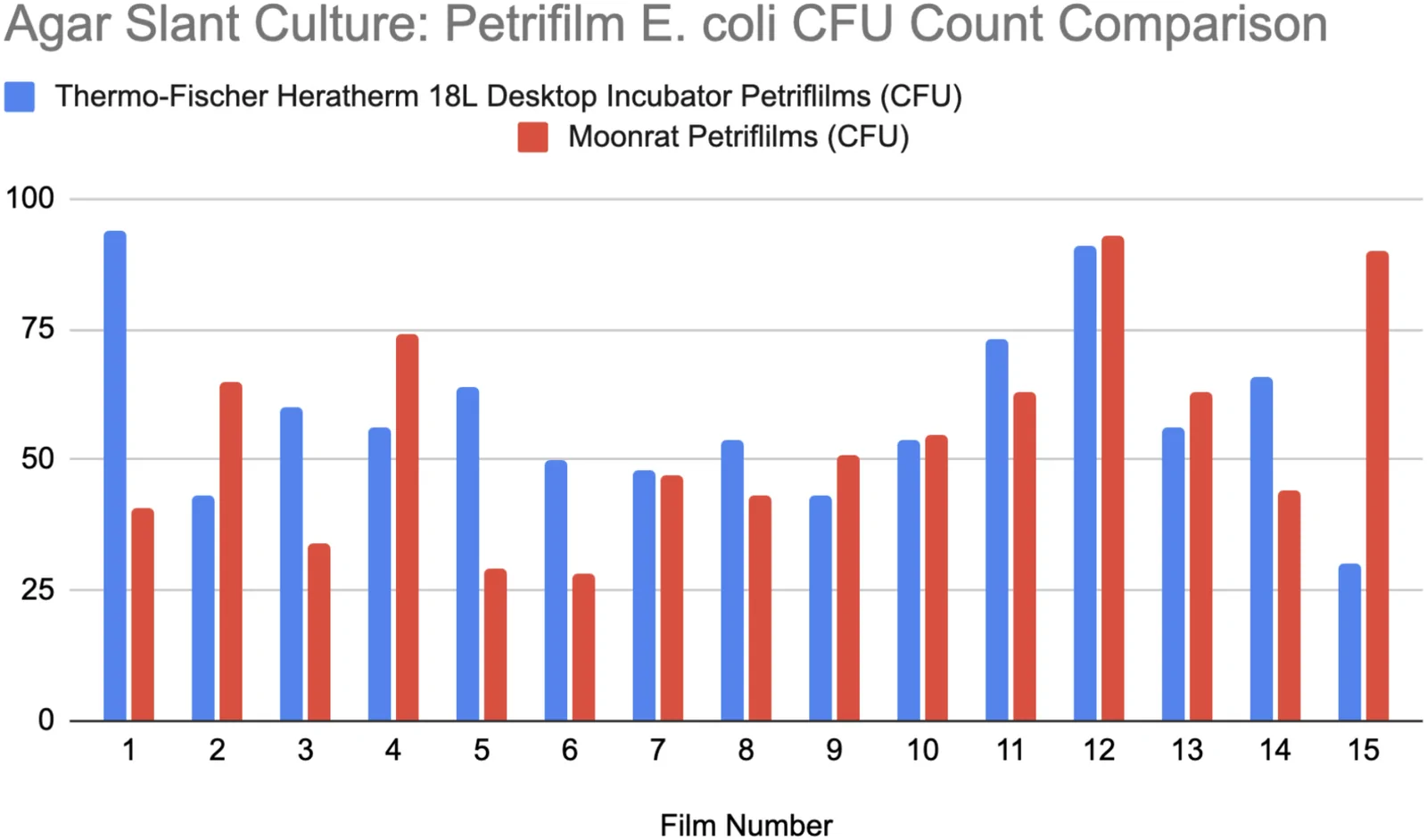
Agar slant comparison.

### 6.8 Safety

There are two possible points of failure for the portable incubator:

• an electrical short and
• thermal runaway.

The Moonrat II printed circuit board assembly contains a 6-amp polyfuse. If a wire broke inside the Moonrat or some other electrical short occurred, this would limit the current draw to 6 amps. This device resets itself automatically when current is removed.

Thermal runaway could in theory be caused by a software error, a sensor failure, or a subtle hardware failure in the heating chamber. A simple short which lowered the resistance would not cause thermal runaway, as the polyfuse would limit the amperage. The Moonrat II software can detect when a wire to the control temperature sensor breaks. When this happens it cuts power to the transistor, blocking further heating. If something goes wrong and the temperature rises, the power will naturally be cut by all three control strategies.

We have no remedy in the unlikely event that the control thermometer returns a reading which does not indicate a disconnect, but nonetheless is inaccurate (that is, it erroneously reports “20°C” when the internal temperature is 40°C or higher). The heating cloth resistance is 12.7 Ω. In this failure mode, 12 V would be supplied continuously to the heating cloths and 11.3 W would be erroneously and continuously added to the Moonrat II, which weighs 1.34 kg. The external temperature becomes “hot to the touch” — like a light bulb — but not hot enough to be generally dangerous. If this happens, the battery would be fully drained after 2 h, and the system would cool to ambient temperature. Future designs will include a thermal fuse in the incubation chamber.

### 6.9 Software architecture

The PID controller is implemented with the widely used PID controller in the Arduino library by Brett Beauregard [17]. The Fuzzy controller is implemented in a separate file. The “bang–bang” thermostat control is so simple it needs no file.

The Moonrat II software follows a typical Arduino “simple loop” or “super loop” architecture. It internally maintains a representation of the state of the menu system on the OLED so that at any time, a button press may be properly processed. The action of this loop is:

1. Check to see if a button has been pressed. If so, process it based on context.
2. Check to see if an “x” or “r” character has been received on the serial port. If so, either dump the temperature vs. time data or reset those samples, respectively.
3. Check to see if the temperature has reached the target to change the anti-windup tuning of the PID controller.
4. If the report time duration has passed:
  • Print a report on the serial monitor for debugging current temperature, hours incubated, etc.
  • Compute the pulse width modulation (PWM) power for the transistor that gates the 12 V power as a function of the current temperature and output that PWM value (the algorithm for this depends on the strategy selected at compile time).
5. If the date record time duration has passed, store the current temperature sample in the EEPROM.

The EEPROM records data persistently, even through loss of power. The EEPROM stores the temperature every minute. This is used to render the graph of temperature against time when the user chooses to display that data. The EEPROM also stores the configured incubation temperature, incubation time, and the remaining incubation time. This is a great convenience to the user.

### 6.10 Conclusion

The Moonrat II is a $225USD fully open-source portable incubator designed for rugged field use. It allows a researcher to perform many kinds of biological tests in the field without needing to transport samples to a laboratory. This will allow rural communities to test their drinking water for contamination, and will support “in the field” scientific study.

## CRediT authorship contribution statement

**Robert L. Read:** Writing – review & editing, Writing – original draft, Validation, Supervision, Software, Investigation, Conceptualization. **Melanie Y. Laporte:** Writing – review & editing, Supervision, Methodology. **Forrest “Lee” Erickson:** Writing – review & editing, Supervision, Project administration, Investigation. **Horacio García–Vázquez:** Writing – original draft, Software, Investigation. **Silvia A. Casillas:** Validation, Investigation. **L. Enrique Ruiz–Fernández:** Methodology, Investigation.

## Declaration of competing interest

The authors declare that they have no known competing financial interests or personal relationships that could have appeared to influence the work reported in this paper.
